# Monitoring bottlenose dolphin leukocyte cytokine mRNA responsiveness by qPCR

**DOI:** 10.1371/journal.pone.0189437

**Published:** 2017-12-22

**Authors:** Amelia Ruth Hofstetter, Kirsten C. Eberle, Stephanie K. Venn-Watson, Eric D. Jensen, Tracy J. Porter, Theresa E. Waters, Randy E. Sacco

**Affiliations:** 1 Ruminant Diseases and Immunology Research Unit, National Animal Disease Center, Agricultural Research Service, United States Department of Agriculture, Ames, Iowa, United States of America; 2 Translational Medicine and Research Program, National Marine Mammal Foundation, San Diego, California, United States of America; 3 United States Navy Marine Mammal Program, San Diego, California, United States of America; Charles University, CZECH REPUBLIC

## Abstract

Both veterinarians caring for dolphins in managed populations and researchers monitoring wild populations use blood-based diagnostics to monitor bottlenose dolphin (*Tursiops truncatus*) health. Quantitative PCR (qPCR) can be used to assess cytokine transcription patterns of peripheral blood mononuclear cells (PBMC). This can supplement currently available blood tests with information on immune status. Full realization of this potential requires establishment of normal ranges of cytokine gene transcription levels in bottlenose dolphins. We surveyed four dolphins over the span of seven months by serial bleeds. PBMC were stimulated with phytohaemagglutinin (1, 5, and 10 μg/mL) and concanavalin A (1 μg/mL) for 48 H in vitro. RNA from these cultures was probed by qPCR using *Tursiops truncatus*-specific primers (IL-1α, IL-1β, IL-1RA, IL-2, IL-4, IL-6, IL-8, IL-10, IL-12p40, IL-13, IL-18, IFN-γ and TNF-α). Two blood samples from an additional bottlenose dolphin diagnosed with acute pulmonary disease add further perspective to the data. We observed that mitogen choice made a significant difference in the magnitude of gene transcription observed. On the other hand, most cytokines tested exhibited limited intra-animal variation. However, IL-6 and IL-12p40 differed between older and younger dolphins. Furthermore, the magnitude of mitogenic response clusters the tested cytokines into three groups. The data provide a reference for the selection of target cytokine mRNAs and their expected range of mitogen-stimulated cytokine gene transcription for future studies.

## Introduction

Veterinary care of dolphins in managed populations has continued to progress with the adoption of advanced medical technologies available in humans and other animal species [1]. Concerns about the effects of anthropogenic pollutants have driven a considerable body of research into the immunotoxicology of marine mammals [2]. In addition, multiple groups have reported on immune responses to naturally-occurring infections of cetaceans, such as with morbillivirus, parasites and bacterial infection [3–6]. Dolphin-specific immunoreagents are forthcoming but still limited, as are studies monitoring animals of known health status over time [7]. While capture and release blood sampling from wild, free-ranging cetaceans has demonstrated signs consistent with physiological stress that may limit the ability to define normal cytokine levels, blood sampling from dolphins under human care can be easily and routinely conducted in-water using trained behaviors (i.e. with little to no stress) [8, 9]. Therefore, it would benefit veterinarians to define immune reference ranges in bottlenose dolphin blood [10].

Cytokines are proteins secreted by immune cells transmitting autocrine, paracrine or endocrine signals. The levels of cytokine production by PBMC provide information on systemic inflammatory trends. We examined thirteen cytokines. TNF-α, IFN-γ and IL-12p40 are pro-inflammatory cytokines [11, 12]. Members of the IL-1 family, including IL-1α, IL-1β, IL-1RA, and IL-18, regulate pro-inflammatory responses [13, 14]. IL-4, IL-13 and IL-10 are generally considered anti-inflammatory and promote T_H_2 responses [11, 15, 16]. IL-6 has pleiotropic impacts depending on context [17]. Finally, IL-2 is a growth factor [18], and IL-8 is a chemoattractant for leukocytes [11]. The selection of cytokines tested in this study covers some of the better–characterized immune mediators, and samples both pro- and anti-inflammatory signaling cassettes.

Real-time quantitative PCR (qPCR) is a powerful tool to measure differences in gene transcripts from a small amount of starting material [19]. qPCR can be used to assess gene transcription levels before protein-based reagents are available [20, 21]. There are two ways to observe cytokine gene transcription, and both provide different information. Cytokine gene transcription can be probed from RNA preserved immediately ex vivo. This has the advantage of reflecting ongoing gene transcription in the subject at the time of the blood draw. Alternatively, gene transcription can be probed from cells that have been stimulated with a mitogen in vitro before RNA collection. This type of study reflects instead the gene transcription potential of lymphocytes, which may be affected by chromatin structure, transcription factors, and gene promoter and repressor availability, for example. Although white blood cells comprise less than 1% of the cellular component of blood [22, 23], they are a highly dynamic population [24] which can be altered by health, stress status, and environment [8, 24]. This method avoids the need for immediate RNA preservation. Both approaches have been used with cetacean samples previously ([21, 25–30]).

We chose to use the latter approach, measuring mitogen-stimulated cytokine gene transcription, or MSCGT. MSCGT levels were previously reported for bottlenose dolphin and harbor porpoise peripheral blood mononuclear cells (PBMC) [21, 25, 26, 28, 30]. However, longitudinal trends have not been reported except in the context of a vaccine challenge [30]. For the dolphin studies, results using only one mitogen were reported [26]. To develop a broader understanding of the normal range of MSCGT in bottlenose dolphins, we examined PBMC collected from dolphins in the U.S. Navy’s Marine Mammal Program (MMP) over 7 months. The common mitogens concanavalin A (Con A) and phytohaemagglutinin (PHA) are known to elicit proliferative responses from dolphin blood lymphocytes [31]. Con A and a range of concentrations of PHA were used to stimulate dolphin PBMC for 48 H in vitro before RNA was collected. By using qPCR, we obtained gene transcription data for 13 different cytokines after four different stimulation conditions from a limited amount of blood, demonstrating the feasibility of using this technique clinically.

Our results underscore the importance of mitogen choice, which impacts the maximal MSCGT in a cytokine-dependent manner. We found surprisingly little intra-animal normal MSCGT variation in ten of the thirteen cytokines. However, we observed increased IL-6 and IL-12p40 MSCGT in older dolphins with chronic illness. We also demonstrate that the cytokines tested cluster into three groups after mitogenic stimulation: highly variable cytokines, moderately variable cytokines, and the IL-1 family plus IL-8, which showed the weakest response to stimulation. These data will be informative in the design of future studies using qPCR to study bottlenose dolphin PBMC MSCGT.

## Materials and methods

### Animal care statement

The U.S. Navy Marine Mammal Program (MMP) houses and cares for a population of dolphins in San Diego Bay, CA. The MMP is AAALAC-accredited and adheres to the national standards of the United States Public Health Service Policy on the Humane Care and Use of Laboratory Animals and the Animal Welfare Act. The MMP’s animal care and use program is routinely reviewed by an institutional animal care and use committee (IACUC) and the Department of Defense Bureau of Medicine. The animal use and care protocol for MMP dolphins in support of this study was approved by the MMP’s IACUC and the Navy’s Bureau of Medicine (IACUC Approval No. 106–2013, BUMED NRD-879). The study was funded by the Office of Naval Research (N0001412IP20029).

### Study design

Four dolphins were monitored over 13 months (May 2014-June 2015) as part of a longitudinal health study aimed at collecting baseline breath metabolomic data [32]. Animal samples were collected monthly for a suite of diagnostics including breath samples, complete blood counts plasma chemistry, and cytokine analysis. Marine mammal veterinarians and research assistants at the MMP conducted routine health assessments, including physical observations and morphologic measurements. This ongoing study provided an opportunity to measure MSCGT in PBMC from dolphin blood. We had access to blood samples from the four dolphins (A, B, C and D) during 7 months of the study (September 2015-May 2015, Table 1). These will be referred to as the “cohort” dolphins. During the study, an additional dolphin was diagnosed with acute pulmonary disease. At this time, we obtained two blood samples from this dolphin (E) collected one week apart. Our goal was to determine the range and variability of MSCGT in dolphin PBMC. These would then serve as baseline MSCGT values against which to compare future dolphin PBMCs, e.g. samples from a dolphin with an abnormal hemogram or behavior, or after an environmental change. Over the course of the seven months we received blood, one of the cohort dolphins was diagnosed with acute pulmonary disease, another had an abnormal, inflammatory hemogram with unknown etiology, and a third became pregnant (Table 1). To determine true MSCGT baseline values, data from these sampling dates were omitted from the analysis unless indicated otherwise. The number of remaining samples for each dolphin is listed in Table 1. These will be referred to as “normal” samples. Note that samples from dolphins with underlying chronic dental disease but no symptoms of acute disease are still considered normal for this study.

**Table 1:** Abbreviated dolphin clinical record.

ID	Age (Y)	Sex	Health	Event Start	Event End	Normal Sample *n*
**A**	35	M	Moderate-severe periodontal disease.	Ongoing	Ongoing	3–4
			Mild inflammatory hemogram. Ultrasound findings consistent with acute pulmonary disease. Animal responsive to antibiotic therapy.	Oct 2014	Dec 2014	
**B**	10	M	Mild inflammatory hemogram—unknown etiology based on physical exam and thoracic ultrasound. No therapy described. Animal noted to be reproductively active during this period.	Aug 2014	Oct 2014	5
			Healthy	Oct 2014	Apr 2015	
**C**	13	F	Healthy (non-pregnant)	Aug 2014	Nov 2014	2
			Healthy (pregnant)	Nov 2014	Apr 2015	
**D**	44	M	Mild-moderate dental disease	Ongoing	Ongoing	4
			Animal noted to be reproductively active during this period.	Jun 2014	Apr 2015	
**E**	26	F	Moderate-severe inflammatory hemogram. Ultrasound findings consistent with acute pulmonary disease. Animal responsive to antibiotic and antifungal therapy.	Sep 2014	Sep 2014	n/a

Age (in years), sex and an abbreviated clinical record for the five dolphins involved in the study are provided. Both chronic and acute health categories are included in the “Health” column. Date of first clinical notation of health status within study period noted in “Event Start” column. Date of last clinical notation of health status within study period noted in “Event End” column. The number of blood samples remaining after samples from during pregnancy, inflammatory hemogram or pulmonary disease were removed is noted in the “Normal Sample *n*” Column.

### Sample collection

Blood was collected in BD Vacutainer Cell Preparation Tubes (CPT) containing Sodium Citrate (BD Biosciences). Tubes were centrifuged at 1500 x g for 30 min. at room temperature before overnight shipment on ice packs. Upon arrival at the USDA laboratory, PBMC were collected from the top of the CPT and washed in PBS followed by centrifugation for 10 min at 1200 RPM. Residual RBCs were lysed by 1 min. incubation in 13.2 cM phosphate buffer followed by restoration into 2.7% saline 13.2 cM phosphate buffer. PBMC were washed in PBS, then re-suspended in 1 mL complete Dulbecco’s Modified Eagle Medium (cDMEM) and counted. Cells were plated at 2 x 10^5^ cells/100 μL/well in sterile 96-well plates. Stimulation was added in 100 μL for a final concentration of 1 μg/mL concanavalin A (Con A, Sigma), or 1, 5 or 10 μg/mL phytohemagglutinin (PHA-P, Sigma; concentrations within a physiologically relevant range [33]). 100 μL cDMEM alone was added for media controls. The cells were incubated for 24 or 48 H at 37°C. However, few samples had sufficient cells for 24 H stimulation, and fewer were from normal samples. Therefore, the 24 H data is included in the appendix (S1 Appendix) but not discussed further.

To collect cells, plates were centrifuged at 1000 RPM for 5 min. Supernatants were removed, and cell pellets were washed in PBS. Cell pellets were collected in 100 μL/well of Qiagen RLT buffer supplemented with 10 μL/mL β-mercaptoethanol. 5–6 technically identical wells were pooled together and frozen at -80°C.

### RNA purification

Qiagen’s RNeasy Mini Kit protocol was followed to purify RNA from PBMC. Briefly, cell pellets in RLT buffer were thawed, then passed through a QIAshredder spin column (Qiagen). One volume 70% ethanol was added before transferring lysate to an RNeasy Mini spin column. Bound RNA was washed in Qiagen RW1 buffer before and after on-column Qiagen DNAse I digestion, followed by two Qiagen RPE buffer washes. RNA was eluted in 50 μL RNAse-free water. RNA was stored at -80°C.

### cDNA synthesis

300 ng Random Primers (Invitrogen) and 2 μL 10 mM dNTP mix (Invitrogen) were added to 20 μL RNA. The resulting mix was heated to 65°C for 5 min. then quick-chilled to 4°C. 8 μL 5X First-Strand Buffer (Invitrogen), 2 μL 0.1M DTT (Invitrogen) and 2 μL Invitrogen Superscript III Reverse Transcriptase were added per 20 μL RNA. cDNA synthesis was conducted by 5 min. incubation at 25°C, followed by 50°C for 1 H and inactivation at 70°C for 15 min. cDNA was diluted 1:10 in nuclease-free water and stored at -80°C.

### Relative real-time PCR

Primers for the 13 cytokines described, as well as RPS9, were designed using Primer 3 (http://bioinfo.ut.ee/primer3-0.4.0) based on bottlenose dolphin sequences obtained from GenBank (Table 2). Primers were synthesized by Integrated DNA Technologies. PCR products were verified by a combination of base pair size and sequencing. PCR reactions were conducted in 20 μL containing 2 μL cDNA template, 1.25 μL each primer at 10 μM, and 10 μL of 2X Applied Biosystems Power SYBR Green PCR Mastermix (ThermoFisher Scientific) in nuclease-free water. PCR was run on an Applied Biosystems 7300 Real Time PCR System under running conditions as in Sitt et al. [26]. At least two technical replicates were used to obtain each average Ct (AvgCt) value.

**Table 2:** List of primers used.

Gene Name	Accession Number	Primer Sequence (5’-3’)	Amplicon Size (bp)
**RPS9 (*T*.*t*.)**	EU638309.1	F: GACGCTGGATGAGAAAGACC	119
		R: TCAGGCCCAAGATGTAATCC	
**IL-1α (*T*.*t*.)**	AB028215.1	F: CAGCTTCCAGAGCAACATGA	129
		R: TTTAATGCAGCAGCCATGAG	
**IL-1β (*T*.*t*.)**	AB028216.1	F: CCCAAAGTGGAAGATGGAAA	84
		R: GGGTACAGGGCAGATTCAAA	
**IL-1RA (*T*.*t*.)**	AB038268.1	F: TGTGGCAAAATGGAAAACAA	108
		R: CCCTTCCAGAAAGGACATCA	
**IL-2 (*T*.*t*.)**	EU638316.1	F: ATGCCCAAGAAGGCTACAGA	106
		R: TCGAGTTCTGGGTTTTGCTT	
**IL-4 (*T*.*t*.)**	AB020732.1	F: TCTCACCTCCCAACTGATCC	125
		R: TTGCTGTGAGGATGTTCAGC	
**IL-6 (*T*.*t*.)**	XM_004330286	F: CCTTCCAAAAATGGCAGAAA	150
		R: TCGATGCTTCCCTTATCACC	
**IL-8 (*T*.*t*.)**	NM_001280642.1	F: CCCTTCCACCCCAAATTTAT	139
		R: CAACCTTCTGCACCCACTTT	
**IL-10 (*T*.*t*.)**	AB775207.1	F: TGTTGAGCCAGTCTCTGCTG	93
		R: GCATCACCTCCTCCAGGTAA	
**IL-12p40 (*T*.*t*.)**	EU638319.1	F: AGATGCTGGGCAGTACACCT	95
		R: CAGTGGACCAGATTCCGTCT	
**IL-13 (*T*.*t*.)**	EU638317.1	F: GCATGGTGTGGAGTGTCAAC	137
		R: AGCTGAGGGCTTGTGAAGAC	
**IL-18 (*T*.*t*.)**	EU638310.1	F: AGTCAACCCGTCTTTGAGGA	62
		R: ATGGTCTGGGGTGCATTATC	
**IFN-γ (*T*.*t*.)**	AB022044.2	F: GCGCAAAGCCATAAGTGAAC	103
		R: TCTCTGGCCTCGAAACAGAT	
**TNF-α (*T*.*t*)**	AB049358.1	F: ACCAGCCAGGAGAGAGACAA	147
		R: CTCAAGTCCAGCTGGGAGAC	

A list of the primers used in the manuscript is provided. Accession numbers are from NCBI’s GenBank. Forward primers are listed after F and reverse primers are listed after R. *T*.*t*.: *Tursiops truncatus*.

### PCR analysis

Relative quantities (RQ) of each cytokine message were calculated using Applied Biosystems Sequence Detection Software v. 1.4 by the equation RQ = 2^(-ΔΔCt)^ where ΔCt = AvgCt using immunomediator primers − AvgCt of RPS9, and ΔΔCt = ΔCt of stimulated cells − ΔCt of mock treated cells. Chen et al. [20] recently demonstrated increased stability of three alternate housekeeping genes over RPS9 when using primers derived from *Stenella coeruleoalba* and *Delphinapterus leucas* on bottlenose dolphin samples. We confirmed this observation with data from the primer sets from Chen et al. [20] and a selection of 24–37 of our cDNA samples using the RefFinder program ([34], [20]; S1 Fig). However, when Ct values for the most stable of the three housekeeping genes, phosphoglycerate kinase 1 (PGK1) were used to normalize our previously obtained ct values for the cytokines, little difference was observed in dolphin phenotypes or trends (Compare Figures A-M in S1 Appendix to Figures N-Z in S1 Appendix). Limited amounts of sample cDNA prevented us from replicating the dataset entirely with PGK1, so we present here the data as originally obtained, using RPS9 as the housekeeping gene. RQ values were graphed with log_2_ y axes using Prism (GraphPad). For each stimulation condition and cytokine, the log_2_ geometric mean RQ value (MeanRQ) was determined by transforming all RQ values by the equation RQ = log_2_RQ, averaging log_2_RQ values from each sampling date per stimulation, then transforming the means by the equation MeanRQ = 2^Avg log2RQ^.

### Statistics

**Fig 1:**
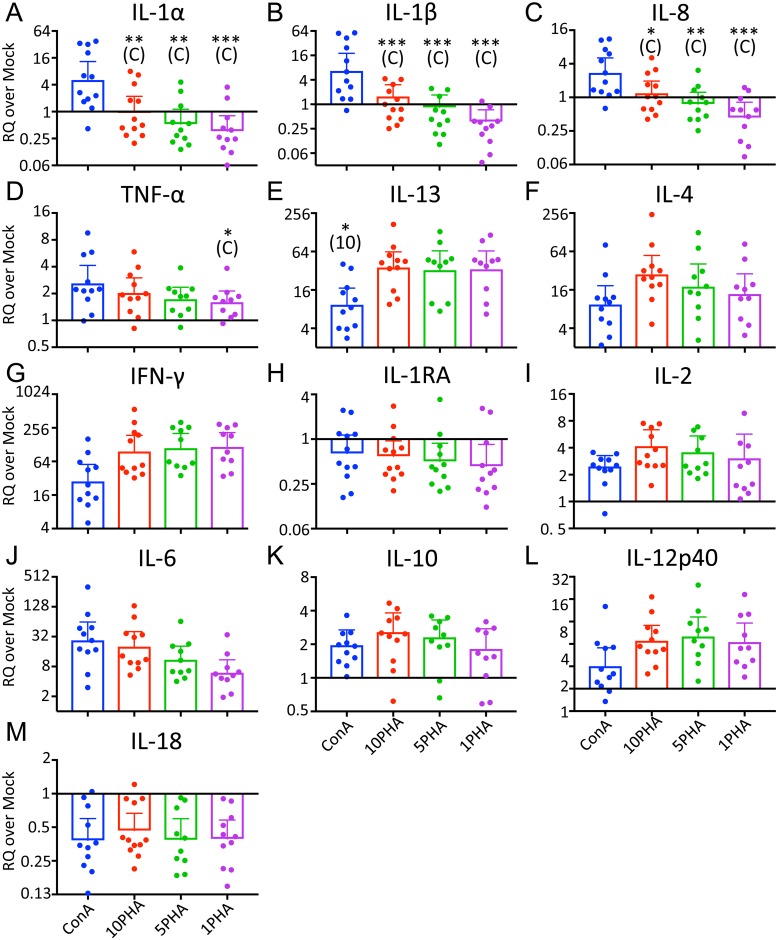
Optimal in vitro stimulant and concentration is cytokine-specific. RQ values obtained from the cohort dolphins for each cytokine are pooled and graphed by stimulus. Bars indicate geometric mean with 95% confidence intervals. Stars indicate a statistically significant difference from stimulus indicated in parentheses (C: Con A, 10: 10 μg/mL PHA) by Tukey’s multiple comparisons test. *: *p* ≤ 00332, **: *p* ≤ 0.0021, ***: *p* ≤ 0.0002.

**Fig 2:**
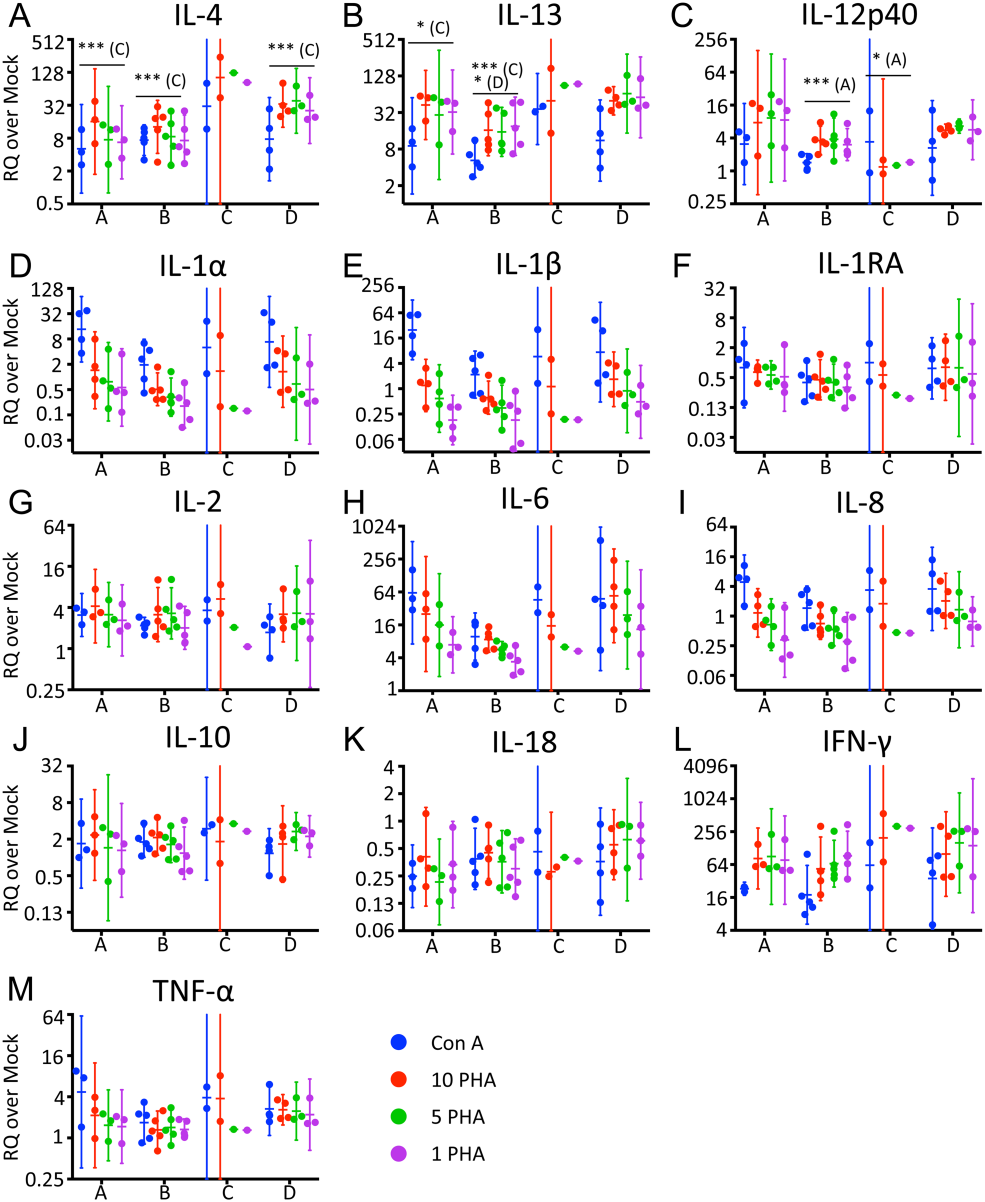
Baseline MSCGT is comparable between dolphins for most cytokines tested. RQ values obtained for each cytokine are shown assorted by each dolphin and stimulus. Error bars depict geometric mean with 95% confidence interval. The error bars for the *n* = 2 data from dolphin C are often cut off due to their relative size compared with the other data sets. Stars indicate a statistically significant difference from dolphin indicated in parentheses (A: dolphin A, C: dolphin C, D, dolphin D) by Tukey’s multiple comparisons test. *: *p* ≤ 00332, ***: *p* ≤ 0.0002.

**Fig 3:**
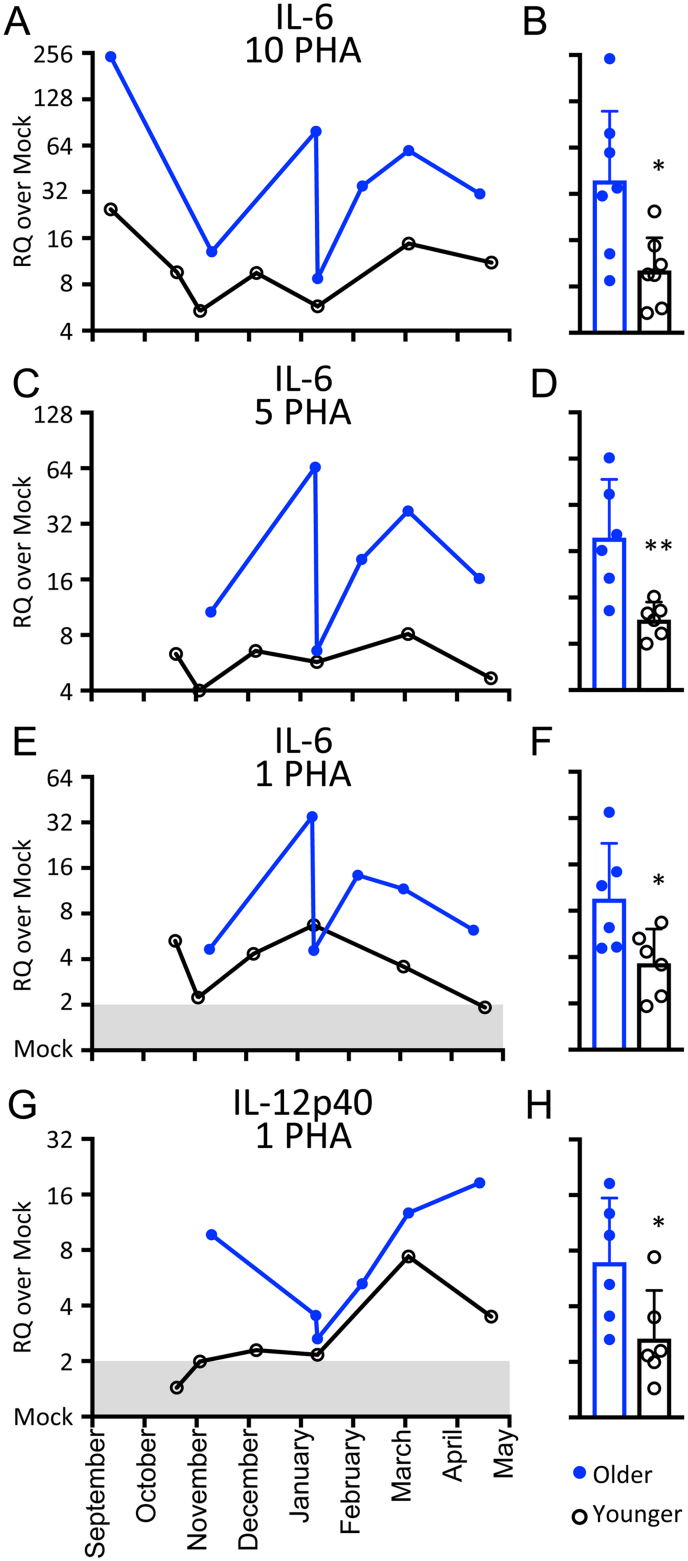
Higher IL-6 and IL-12p40 MSCGT in older dolphins with chronic disease. RQ values for each cytokine and stimulus were pooled into two groups. Three to four samples from dolphin A were pooled with 4 samples from dolphin D. Similarly, 5 samples from dolphin B were pooled with one to two samples from dolphin C. A, C, E, G: RQ values are plotted against sampling date. Shaded area indicates range from 0.5–2 RQ considered no different from mock. B, D, F, H: Bars indicate geometric mean with 95% confidence interval for the same data points as in A, C, E and G. Stars indicate a statistically significant difference between the groups by two-tailed Mann-Whitney test. *: *p* ≤ 00332, **: *p* ≤ 0.0021.

**Fig 4:**
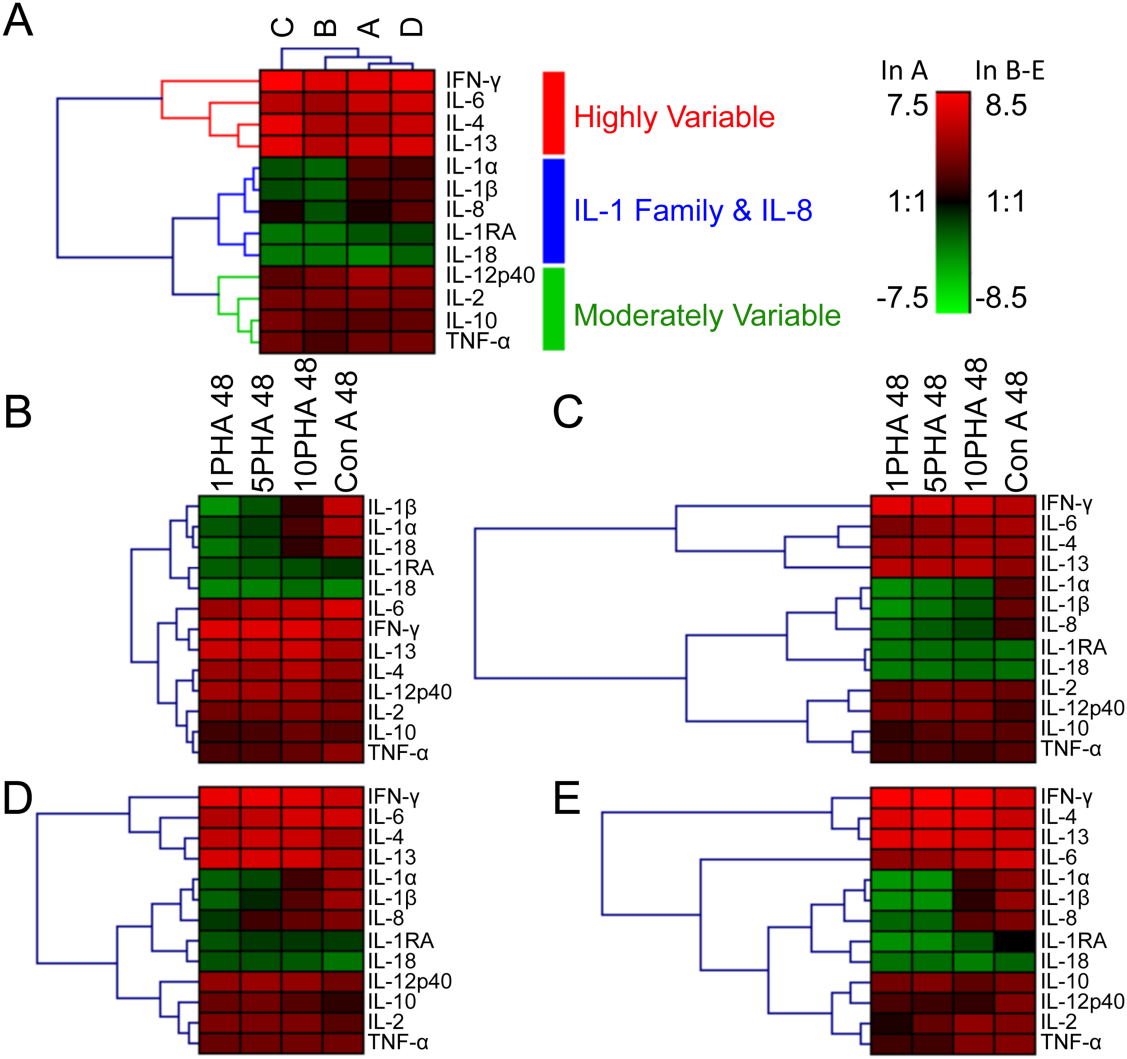
Hierarchical clustering of cytokine responses. MeanRQ values for each cytokine are plotted in heat maps. (A) MeanRQ values for each cytokine were averaged across the four stimulation conditions. Data were clustered by cytokine and dolphin. (B-E) MeanRQ values are plotted for each dolphin. Data were clustered by cytokine. *(B)* Dolphin A; *(C)* dolphin B; *(D)* dolphin D; *(E)* dolphin C. MeanRQ values log_2_ transformed before plotting. Hierarchical clustering conducted using Genesis Software (http://genome.tugraz.at/genesisclient/genesisclient_description.shtml). The Log_2_ legend is to the right of (A); the scale for (A) is on the left and the scale for B-E on the right.

**Fig 5:**
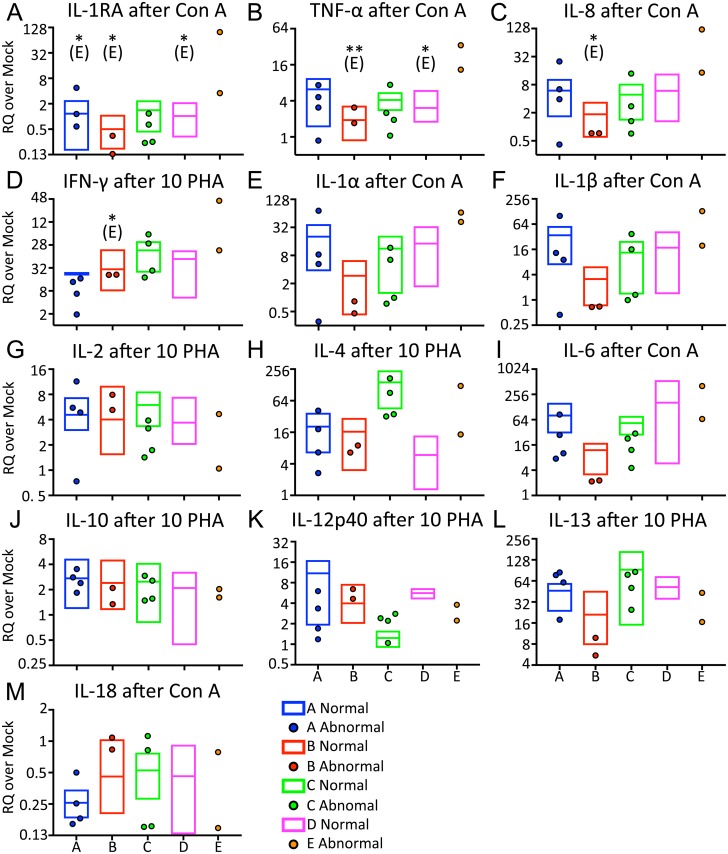
Overlay of abnormal and normal data from each dolphin. A minimum-to-maximum bar represents the range of RQ values obtained for each cytokine from each dolphin’s normal samples. The mean of RQ values from the normal samples is represented by a horizontal line. Overlaid bullet points indicate RQ values from abnormal samples. RQ values were log_2_ transformed and analyzed one-way ANOVA. Stars indicate a statistically significant difference between normal samples from cohort dolphins and the abnormal dolphin E samples by Sidak’s multiple comparisons test. *: *p* ≤ 00332, **: *p* ≤ 0.0021.

For Figs 1 and 2, RQ values from each normal sample were analyzed irrespective of sampling date. Two-way ANOVA was conducted, with Tukey’s multiple comparison post-tests to compare the contribution of stimulus (Fig 1) or dolphin (Fig 2) to the overall variation. For Fig 3, the mean of the RQ values from both whales per group was compared with the mean from the other group by two-tailed Mann-Whitney test. For Fig 4, heat maps were generated by importing the MeanRQ values into Genesis software (Graz University, [35]). The data was log_2_ transformed and hierarchical clustering analysis was performed with average linkage clustering to show relationships between cytokine transcription and dolphins. For Fig 5, RQ values were log_2_ transformed and analyzed by one-way ANOVA, with Sidak’s multiple comparisons post test.

## Results

### Optimal in vitro stimulant and concentration is cytokine-specific

Mitogen choice affects the magnitude of the MSCGT, depending on the cytokine. We tested both 1 μg/mL Con A, as previously reported [26], as well as three concentrations of PHA (1, 5 and 10 μg/mL). Note that cells were stimulated with Con A and 10 μg/mL PHA at each sampling date, but the lower doses of PHA were not always used. As shown in Fig 1A–1C, 1 μg/mL Con A is a significantly better mitogen than any tested dose of PHA for IL-1α, IL-1β, and IL-8. Con A also stimulated higher TNF-α transcription than 1 μg/mL PHA (Fig 1D). However, 10 μg/mL PHA outperformed Con A for transcription of IL-13 (Fig 1E). 2-way ANOVA indicates that the stimulus contributed to the variation of IL-4 and IFN-γ (*p* = 0.0145 and *p* = 0.0295, respectively; Fig 1F and 1G), although Tukey’s multiple comparisons post-test did not identify significant differences between individual treatments. MSCGT of the remaining cytokines was not affected by stimulus according to these analyses.

### MSCGT is comparable between animals for most cytokines tested

We hypothesized that inter-animal variability would cause the baseline MSCGT to differ between the four dolphins. Using the same dataset from Fig 1, we queried differences between the four dolphins using Tukey’s multiple comparisons test. Due to the limited data available, comparisons with dolphin C are inconclusive. However, dolphin C appeared to have increased IL-4 MSCGT compared with the other three dolphins (Fig 2A; *p*<0.0001). Dolphin C also appeared to have increased IL-13 MSCGT over dolphins A and B (Fig 2B; *p* = 0.0308 and *p* = 0.0008, respectively), and decreased IL-12p40 MSCGT compared with dolphin A (Fig 2C; *p* = 0.0377). Aside from dolphin C, we observed higher IL-13 MSCGT in dolphin D than dolphin B (Fig 2B; *p* = 0.0148), and higher IL-12p40 MSCGT in dolphin A than dolphin B (Fig 2C; *p* = 0.0041). The ten remaining cytokines did not show any significant difference in baseline MSCGT between dolphins.

### IL-6 and IL-12p40 MSCGT increased in older dolphins with chronic disease

Two of the dolphins in our study were young (10 and 13 years) and two were of advanced age for the species (35 and 44 years). The two older dolphins had chronic dental disease. We grouped the dolphins by age and graphed the data longitudinally. S2 Appendix shows data from 11 of the 13 cytokines, taken from the stimulation (Con A or 10 μg/mL PHA) that provided the greatest magnitude MSCGT (Fig 1). These cytokines showed no difference between the young and old dolphins regardless of mitogen type or dose. However, the mean IL-6 MSCGT in PBMC from the older dolphins was increased as compared with the younger dolphins at any dose of PHA (Fig 3A–3F). The lowest dose of PHA also revealed increased mean IL-12p40 MSCGT in the older dolphins (Fig 3G and 3H).

### Heirarchical clustering sorts cytokines into three groups

To obtain a global view of the data, the geometric mean of the log_2_ RQ values for all normal sampling dates and stimulations was calculated per dolphin. Using this data set, hierarchical clustering distributes twelve of the thirteen cytokines into three groups (Fig 4A). The first group, “Highly Variable” is comprised of the cytokines with the greatest magnitude MSCGT: IFN-γ, IL-4, IL-13, and IL-6. The remaining cytokines display smaller increases or even decreases in mRNA levels after stimulation. The IL-1 family members all cluster together, along with IL-8. This group contains all the cytokines where decreases in MSCGT relative to unstimulated samples were routinely observed. The final group, “Moderately Variable”, includes TNF-α IL-12p40, IL-2 and IL-10. These cytokines had consistent increases in MSCGT, but to a lower magnitude than the first group. This pattern is largely reproduced in hierarchical clustering for each dolphin (Fig 4B–4E), with a couple exceptions. In samples from dolphin A, IL-4 clustered with the other Moderately Variable cytokines. In samples from dolphin C, IL-6 was less similar to the Highly Variable cytokines, although it did not cluster with the other two groups. In both cases, lower MSCGT levels led to IL-4 or IL-6 failing to cluster with the other Highly Variable cytokines. Therefore, three types of response to stimulation were observed among the cytokines tested: large increases in mRNA levels (“Highly Variable”), moderate increases in mRNA levels (“Moderately Variable”) or the low-magnitude increases or decreases in mRNA levels displayed by the IL-1 family and IL-8.

Within each cluster, the relationships between cytokines typically varied among the dolphins. However, IL-1RA and IL-18 always clustered most tightly together. These were the only two cytokines consistently displaying a decrease in MSCGT relative to unstimulated cells after any tested stimulus (Figs 1H, 1M and 4A–4E).

### Samples from dolphin E have a higher mean MSCGT for selected cytokines

To determine whether the abnormal values that were excluded from the above analyses also deviated from the normal range determined for each cytokine, we compared the RQ values obtained for each cytokine from each dolphin’s abnormal samples to the normal samples for that dolphin. This is presented graphically in Fig 5. Only data generated using the stimulus (Con A or 10 μg/mL PHA) that generated the highest RQ values (Fig 1) is shown. Abnormal and normal RQ values obtained using each stimulus were compared for dolphins A, B, and C by two-tailed Mann-Whitney test. No differences were observed between the abnormal and normal samples for any cytokine. In addition, after log_2_ transformation, RQ values of samples from Dolphin E were compared with the normal samples from the other four dolphins by one-way ANOVA with Sidak’s multiple comparisons post-test. By this analysis, the mean RQ of IL-1RA MSCGT from dolphin E samples was significantly higher than dolphins A (*p* = 0.0342), B (*p* = 0.0053) and D (*p* = 0.0318), but only after Con A stimulation (Fig 5A). Mean TNF-α MSCGT from dolphin E samples was significantly higher than the samples from dolphin B after most stimuli tested (Fig 5B; Con A: *p* = 0.0047; 10 μg/mL PHA: 0.0322; 5 μg/mL PHA: 0.0468) and higher than D after Con A stimulation (*p* = 0.0313). Dolphin E also had higher mean MSCGT than dolphin B for IL-8 for most stimuli tested (Fig 5C; Con A: *p* = 0.021, 10 μg/mL PHA: *p* = 0.0385; 5 μg/mL PHA: *p* = 0.0347) and higher IFN-γ MSCGT in some cases (Fig 5D; Con A: *p* = 0.0299; 10 μg/mL PHA: *p* = 0.0275).

## Discussion

In this study, we used qPCR to establish baseline ranges of MSCGT in bottlenose dolphin PBMC. Previous studies have used qPCR, microarrays and transcriptomic analyses to query whole dolphin and porpoise blood for RNA [6, 8–10, 20, 24, 28], but changes in leukocyte composition of the blood have a significant impact on the whole blood transcriptome [24]. Furthermore, qPCR has increased sensitivity over microarrays [19]. We expanded on previous qPCR studies by acquiring multiple samples from five dolphins over the course of seven months, using four different stimulation conditions, and analyzing five bottlenose dolphin cytokines that have not previously been measured by qPCR. This manuscript provides 14 primers derived from GenBank *T*. *truncatus* cytokine sequences. We show that when analyzing MSCGT, the type and concentration of mitogen used must be informed by the cytokine(s) of interest. There was less intra-dolphin baseline MSCGT variation than expected. However, older dolphins with chronic dental disease had higher IL-6 and IL-12p40 MSCGT. The magnitude of MSCGT clusters the cytokines into three groups: highly variable, moderately variable and the IL-1 family plus IL-8. Finally, the data from dolphin E suggests that TNF-α, IL-1RA, IL-8 and IFN-γ should be compared between larger cohorts of dolphins during clinically normal and abnormal samplings. This report lays the groundwork for future qPCR-based studies of cytokine responses in dolphin PBMC.

The difference in MSCGT profiles between Con A- and PHA-stimulated cells was dramatic. IL-1α, IL-1β and IL-8 had increased MSCGT after Con A stimulation, but often decreased MSCGT after PHA stimulation. In contrast, MSCGT was increased more by PHA stimulation than Con A for IL-13, IL-4 and IFN-γ. The response patterns of TNF-α and IL-6 favored Con A, while those of IL-2 and IL-12p40 favored PHA. The differential cytokine profiles of Con A and PHA are likely related to the cross-linking of different cell surface receptors. PHA binds *N*-acetylglucosamine linked to mannose, while Con A binds α-Mannose, α-Glucose and α-GlcNac [33, 36]. Although both Con A and PHA are thought to cross-link the T cell receptor [37], there is also evidence that they may act as TLR ligands [38]. Con A and PHA have been previously shown to have differential effects on cytokine production in a time-dependent manner [39]. These results underscore the importance of pilot studies to determine the optimal mitogen, dosage, and stimulation time depending on the target cytokine. As demonstrated by the grouped dolphin data for IL-12p40 (Fig 3G and 3H), some differences may be more apparent with lower mitogen concentrations.

We observed fewer than expected differences in baseline MSCGT between dolphins. There was a wide variation in dolphin ages. Dolphins A, B, and D were male, while C was female. However, once the abnormal timepoint samples, including samples from C during pregnancy, were omitted, intra-dolphin differences were only observed for IL-4, IL-13 and IL-12p40. Little weight can be given to the variation between dolphin C and the other three cohort dolphins because of the limited number of normal samples remaining. However, it is interesting to note that IL-4, IL-13 and IL-12 are increased in females of various species [40–42]. Furthermore, the two samples from the female E with acute pulmonary disease trend more like the female dolphin C for IL-4 and IL-12p40 MSCGT than the three male dolphins (Fig 5H and 5K). More samples from female dolphins would clarify the impact of sex on baseline MSCGT in bottlenose dolphins. The only other differences observed in baseline MSCGT between dolphins were lower IL-12p40 and IL-13 MSCGT from dolphin B compared with dolphin A and dolphin D, respectively. Dolphins A, B, and D are all males, but B is the only young male and he didn’t have chronic dental disease. Therefore, age and lack of chronic disease may explain the lower cytokine MSCGT of dolphin B. The majority of cytokines tested did not differ between dolphins when sampled in normal health status. This is one necessary characteristic of clinically useful biomarkers, i.e. intra-animal stability in normal health status despite variables of sex and age.

Our ability to compare MSCGT between dolphins was hindered by the number of abnormal samples that had to be removed from the dataset. As an alternative way to look at the data, the two older dolphins were grouped, as were the two younger dolphins. This provided a larger *n* for comparison, while reflecting age differences and isolating the two dolphins with chronic dental disease. This comparison revealed increased IL-6 and IL-12p40 in the older dolphins with chronic dental disease. IL-6 has previously been identified as a marker of subclinical infection in cetaceans [43] but it is unclear if this is the case for harbor porpoises [9, 10]. IL-12 is a pro-inflammatory cytokine [12]. IL-12 was recently reported as a cytokine transcript elevated in the blood of a wounded beluga whale [29]. Importantly, IL-12p40 not only forms heterodimers with p35 to form the IL-12 cytokine, but also forms heterodimers with p19 to form the IL-23 cytokine. Like IL-12, IL-23 is pro-inflammatory, but is critical for Th17 responses. Furthermore, IL-12p40 can form homodimers that are IL-12 antagonists [12]. It is unclear which type of dimer is most affected by the changes in IL-12p40 transcripts we measured. Elevated IL-12p40 or IL-6 may be due to the age of the dolphins or their chronic disease. As age and chronic illness are linked, both probably contribute to the effect.

Hierarchical clustering revealed three cytokine clusters relating to MSCGT levels. IFN-γ, IL-4, IL-13, and IL-6 typically had the greatest increase in MSCGT. This corroborates previous findings by Sitt et al. where IFN-γ, IL-4 and IL-13 were among the top four cytokines with the greatest stimulation index after Con A treatment [26]. In the two cases where IL-4 (in Dolphin A) and IL-6 (in Dolphin C) did not fall into these clusters, this is explained by a lower magnitude response to mitogenic stimulation. In the case of Dolphin A, IL-4 clustered instead with the moderately variable cytokines. In the case of Dolphin C, IL-6 had lower transcription levels than IFN-γ, IL-4, and IL-13, but not as low as TNF-α, IL-2, IL-10 and IL-12p40. Consequently, it did not fall into any of the three clusters. Interestingly, IL-6 is also the only one of these four cytokines that responded better to ConA stimulation than PHA (Figs 1E, 1F, 1G, 1J and 4). IFN-γ is a pro-inflammatory cytokine produced by Th1 cells and cytotoxic T cells [15]. IL-4 and IL-13 are Th2 cytokines that are both activated downstream of STAT6 [44]. These important T cell cytokines were likely stimulated by PHA and Con A cross-linking the TCR [33, 37, 45].

The moderately variable cluster of cytokines comprises four cytokines all known to regulate IFN-γ production [46, 47]. These cytokines typically had a relatively small increase in mRNA after stimulation, or remained unchanged. Increased blood IL-10 mRNA has been associated with poor health status and increased stress hormones in harbor porpoises [6, 28]. In our study, IL-10 MSCGT showed little variation between dolphins, regardless of disease status (Figs 2J and 5J). T cells can produce TNF-α, IL-2 and IL-10, although monocytes are the major producers of TNF-α [15]. IL-12p40 is primarily produced by antigen presenting cells rather than T cells; the increase in IL-12p40 mRNA that is observed may be due to indirect activation of APC via IFN-γ [15, 48].

The IL-1 family clustered with IL-8 by hierarchical analysis. IL-1α is produced by a wide range of cells but tends to act locally, while IL-1β is primarily produced from monocytes, macrophages and dendritic cells and has paracrine activity [49]. IL-1 family members induce transcription of their own genes while mediating transcription and stabilization of IL-8 and IL-6 mRNAs [13]. IL-1RA is a negative-feedback receptor antagonist for IL-1 [50]. Therefore, correlated responses among IL-1, IL-1RA, and IL-8 are expected. IL-8, IL-1α and IL-1β were previously shown to be up-regulated in response to capture-release stress in bottlenose dolphins [8]. IL-18 is constitutively expressed from macrophages, dendritic cells and epithelial cells, but requires cleavage by caspase-1 to be activated. This may explain the lack of increase in IL-18 MSCGT (Fig 1M).

Determining normal ranges of cytokine gene transcription is most useful if abnormal cytokine levels correlate with abnormal health status. With limited data from dolphins diagnosed with acute pulmonary disease, we can only speculate to the utility of the tested cytokines as biomarkers. However, TNF-α, IL-1RA, IL-8 and IFN-γ were four cytokines with higher MSCGT in dolphin E than in the normal samples from one or more of the cohort dolphins (Fig 5). It is interesting to note that none of these four cytokines demonstrated intra-cohort dolphin variability (Fig 2). Studies are ongoing to analyze TNF-α, IL-1RA, IL-8 and IFN-γ expression in a larger cohort of dolphins.

In this study we present the MSCGT profiles for four dolphins over a seven-month study period, with limited data from an additional dolphin diagnosed with pulmonary disease. The utility of qPCR for performing this type of analysis is displayed by the breadth of data we present from a limited amount of blood provided in each sample. The results provide important information for design of future studies. This includes the relationship between mitogen choice and maximal MSCGT, the baseline range of normal dolphin PBMC cytokine response profiles to two commonly used mitogens, and potential roles of sex and age in MSCGT. Furthermore, this study highlights six cytokines that deserve further exploration with the goal of bottlenose dolphin biomarker discovery: IL-12p40, IL-6, TNF-α, IL-1RA, IL-8 and IFN-γ. This information will be used as a starting point for both immunological and clinical studies using dolphin PBMC in the future.

## Supporting information

S1 Appendix Longitudinal profiles of MSCGT normalized to RPS9 or PGK1. RQ values plotted for each blood collection time over the 7 months of the study. Shaded area indicates a range from 0.5–2 RQ considered no different from mock. Figure A-M, data normalized to RPS9. Figure N-Z, data normalized to PGK1. For each Figure: (1) 24 H incubation with 1 μg/mL PHA; (2) 48 H incubation with 1 μg/mL PHA; (3) 24 H incubation with 5 μg/mL PHA; (4) 48 H incubation with 5 μg/mL PHA; (5) 24 H incubation with 10 μg/mL PHA; (6) 48 H incubation with 10 μg/mL PHA; (7) 24 H incubation with 1 μg/mL Con A; (8) 48 H incubation with 1 μg/mL Con A.
(PPTX)

S2 Appendix Data from normal timepoints, grouped. Normal RQ values for each cytokine and stimulus were pooled into two groups. Three to four samples from dolphin A were pooled with 4 samples from dolphin D. Similarly, 5 samples from dolphin B were pooled with one to two samples from dolphin C. RQ values normalized to RPS9 are plotted against sampling date. Shaded area indicates range from 0.5–2 RQ considered no different from mock.
(PPTX)

S1 Fig Results from RefFinder analysis of four housekeeping genes. The three housekeeping genes determined to be most stable by Chen, et al. [20] (PGK1, RPL4 and HPRT) were compared with RPS9. The primers described by Chen, et al. [20] were used for PGK1, RPL4 and HPRT. Our own RPS9 primers were used for RPS9 (Table 2). The CtCorr (described in [20]) for these genes were determined for 24–37 randomly selected cDNA samples. The CtCorr values were compared using the free online program RefFinder (http://leonxie.esy.es/RefFinder/?type=reference#). This program uses four different computational programs to rank and weight the stability of housekeeping genes. RefFinder then calculates the geometric mean of the weights given by the four programs. The resulting geometric means and geometric standard deviations of the ranking values for each housekeeping gene are shown.
(TIF)
