# Supplementary material for: Monitoring bottlenose dolphin leukocyte cytokine mRNA responsiveness by qPCR
Source: PLoS One. 2017 Dec 22;12(12):e0189437. doi: 10.1371/journal.pone.0189437 (PMC5741220; doi:10.1371/journal.pone.0189437)
Supplement: S1 Appendix — RQ values plotted for each blood collection time over the 7 months of the study. Shaded area indicates a range from 0.5–2 RQ considered no different from mock. Figure A-M, data normalized to RPS9. Figure N-Z, data normalized to PGK1. For each Figure: (1) 24 H incubation with 1 μg/mL PHA; (2) 48 H incubation with 1 μg/mL PHA; (3) 24 H incubation with 5 μg/mL PHA; (4) 48 H incubation with 5 μg/mL PHA; (5) 24 H incubation with 10 μg/mL PHA; (6) 48 H incubation with 10 μg/mL PHA; (7) 24 H incubation with 1 μg/mL Con A; (8) 48 H incubation with 1 μg/mL Con A. (PPTX) [file pone.0189437.s001.pptx]

## Slide 1
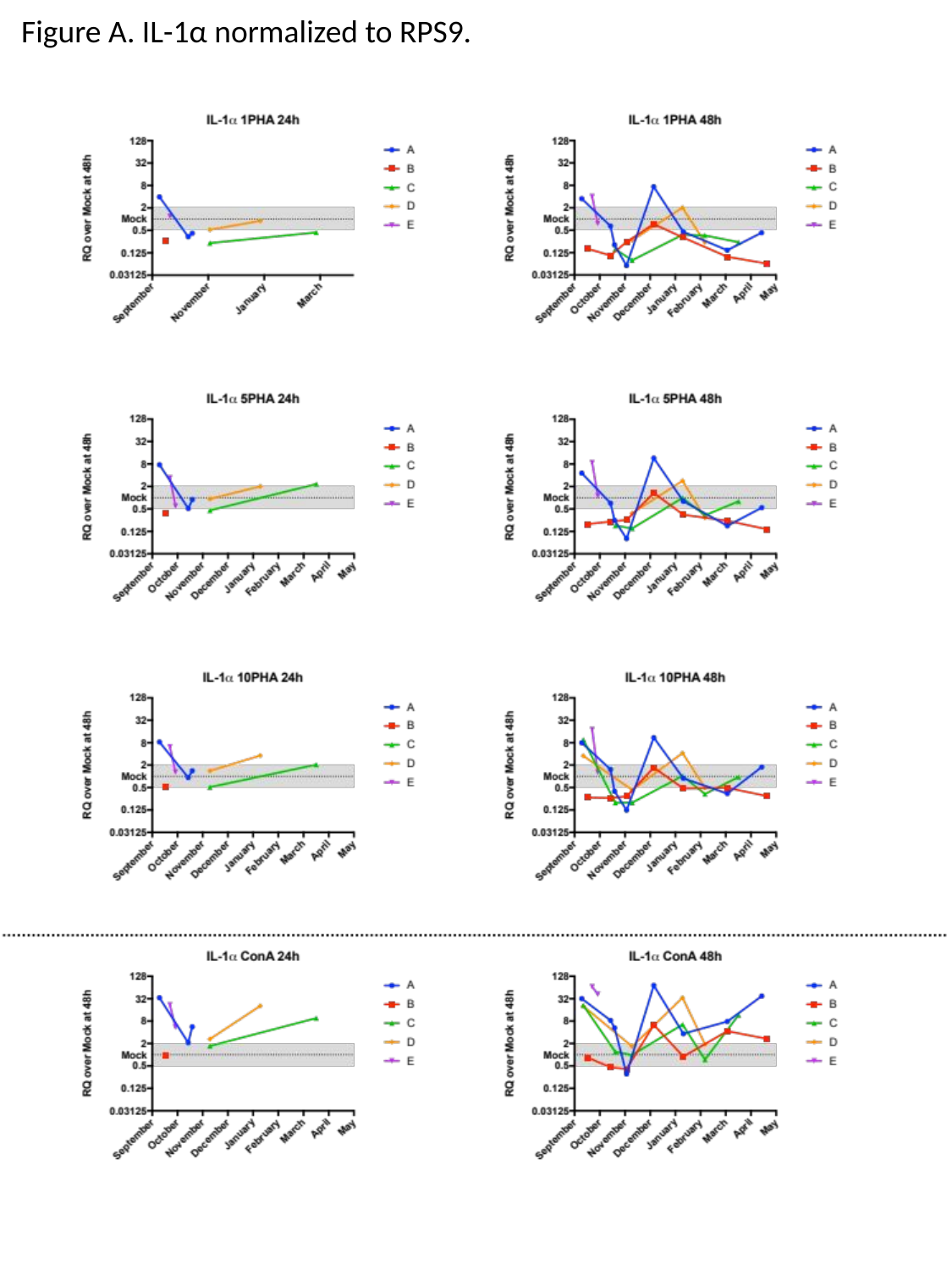

Figure A. IL-1α normalized to RPS9.

## Slide 2
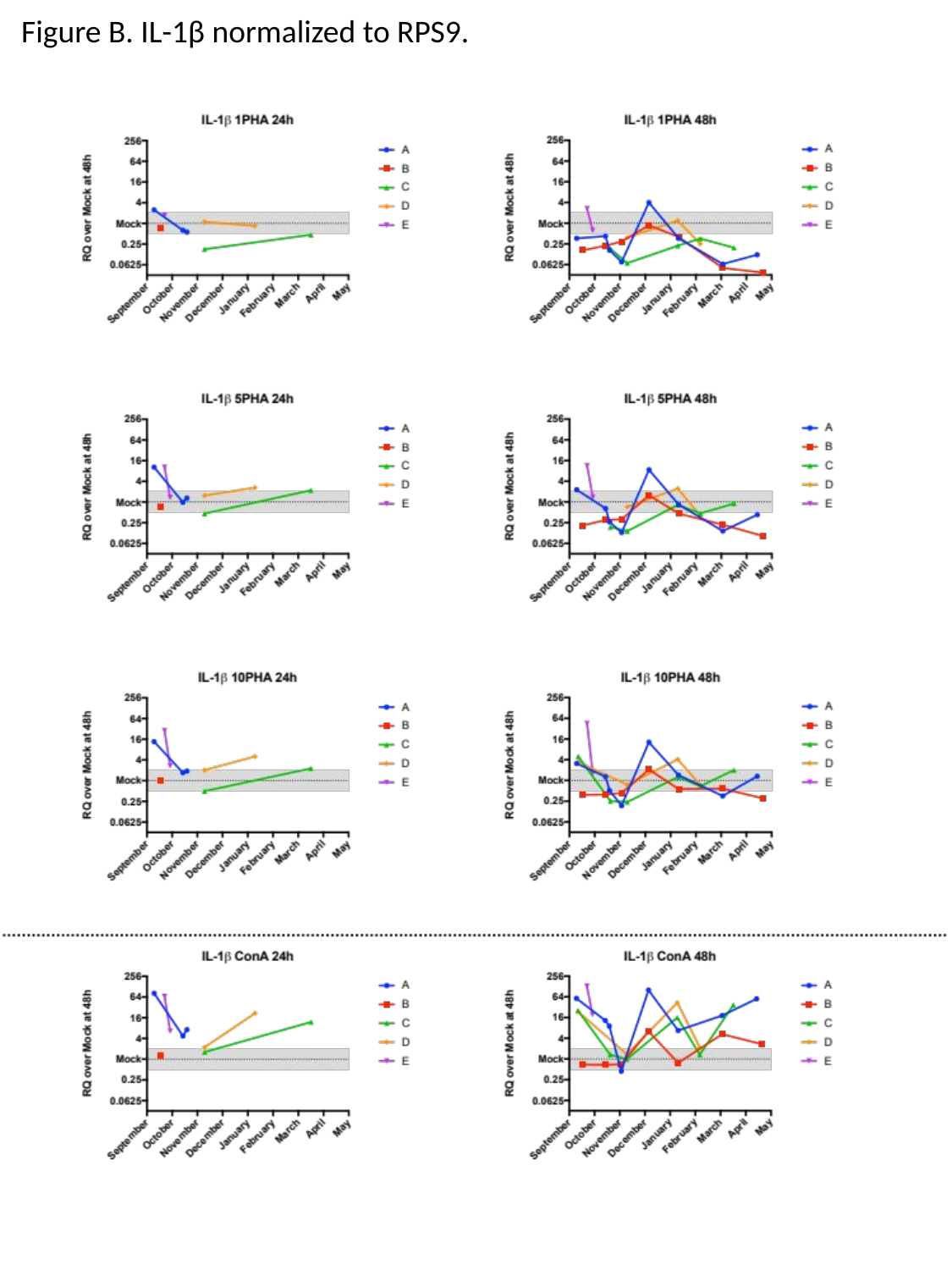

Figure B. IL-1β normalized to RPS9.

## Slide 3
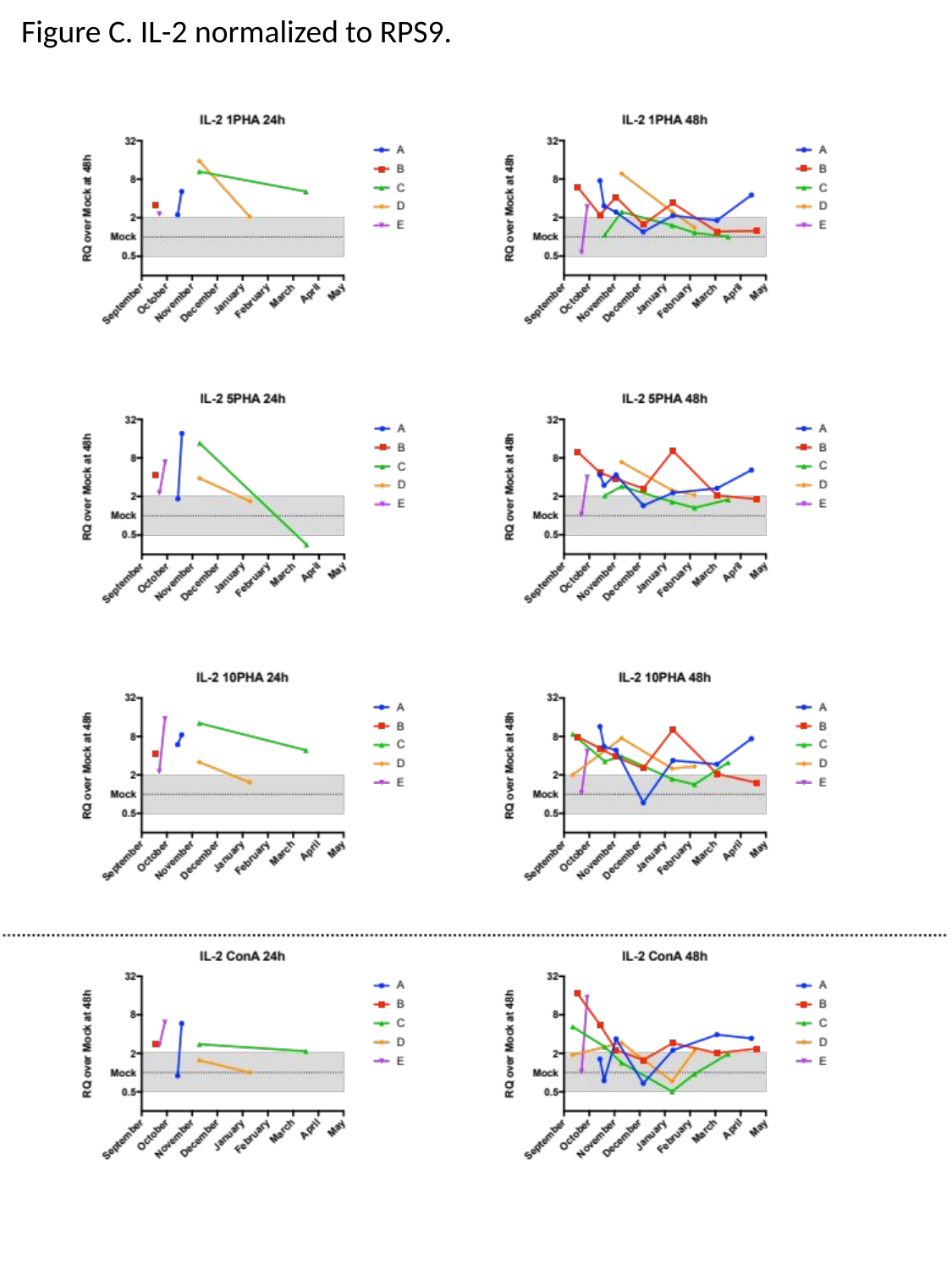

Figure C. IL-2 normalized to RPS9.

## Slide 4
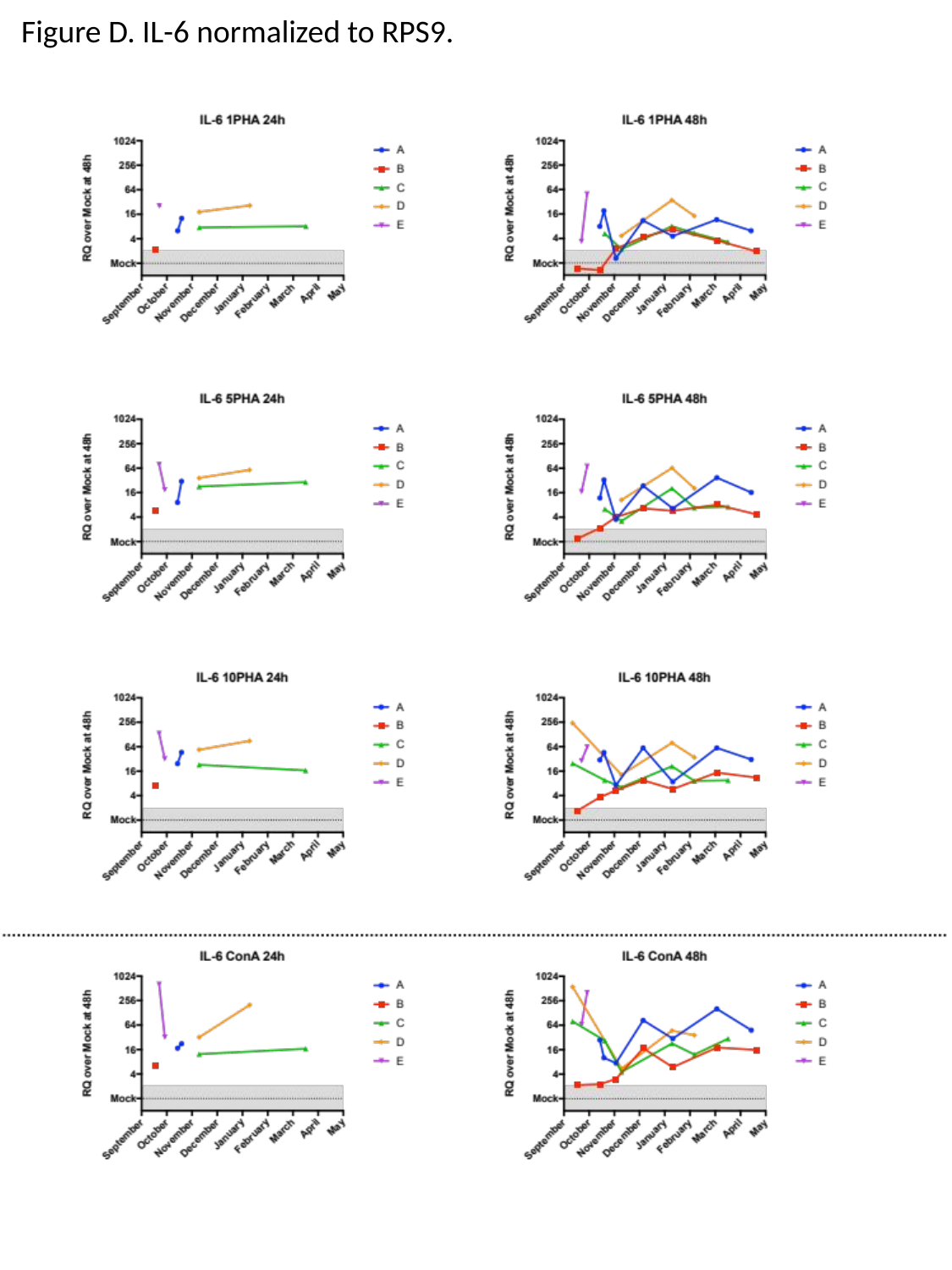

Figure D. IL-6 normalized to RPS9.

## Slide 5
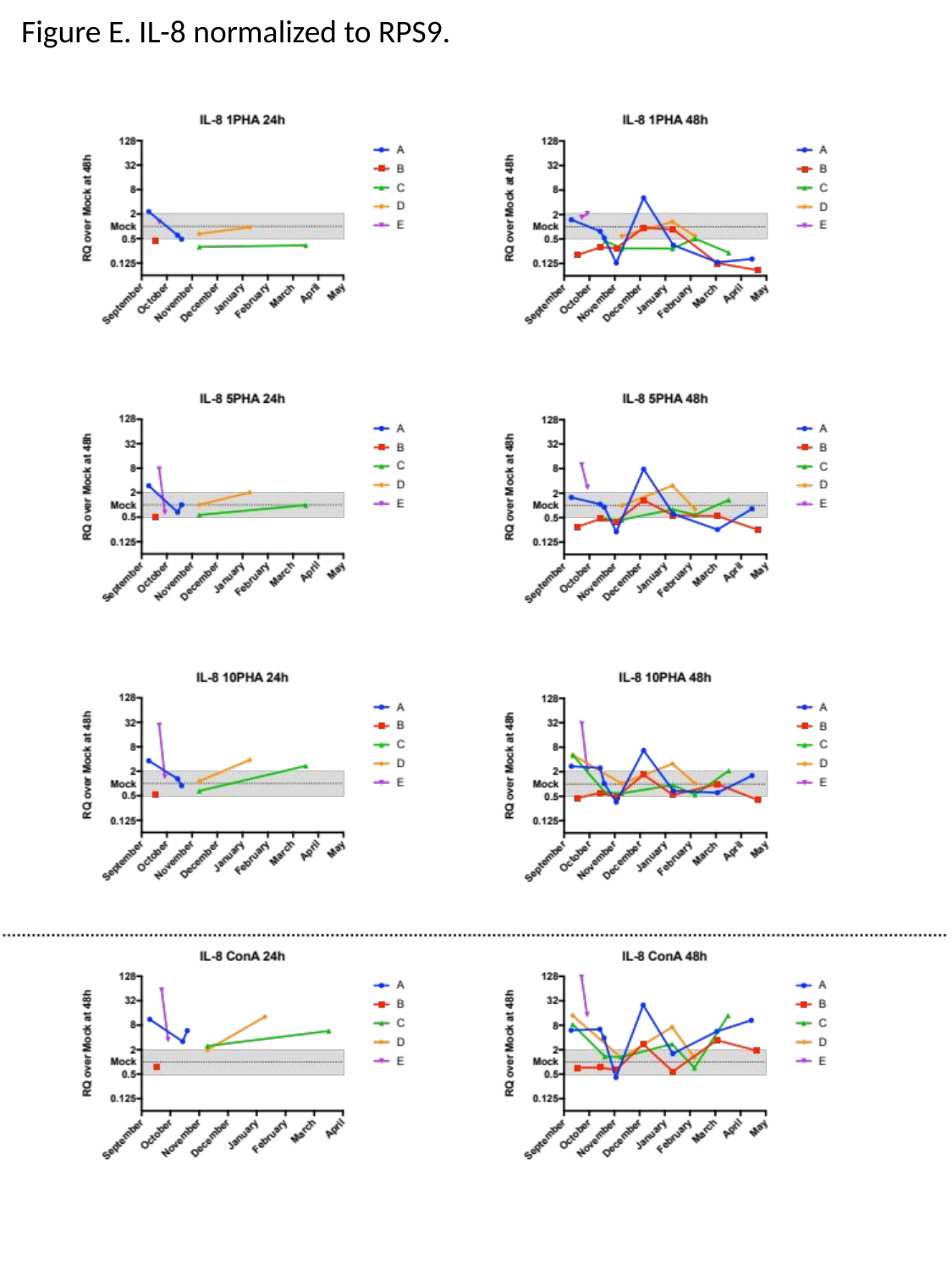

Figure E. IL-8 normalized to RPS9.

## Slide 6
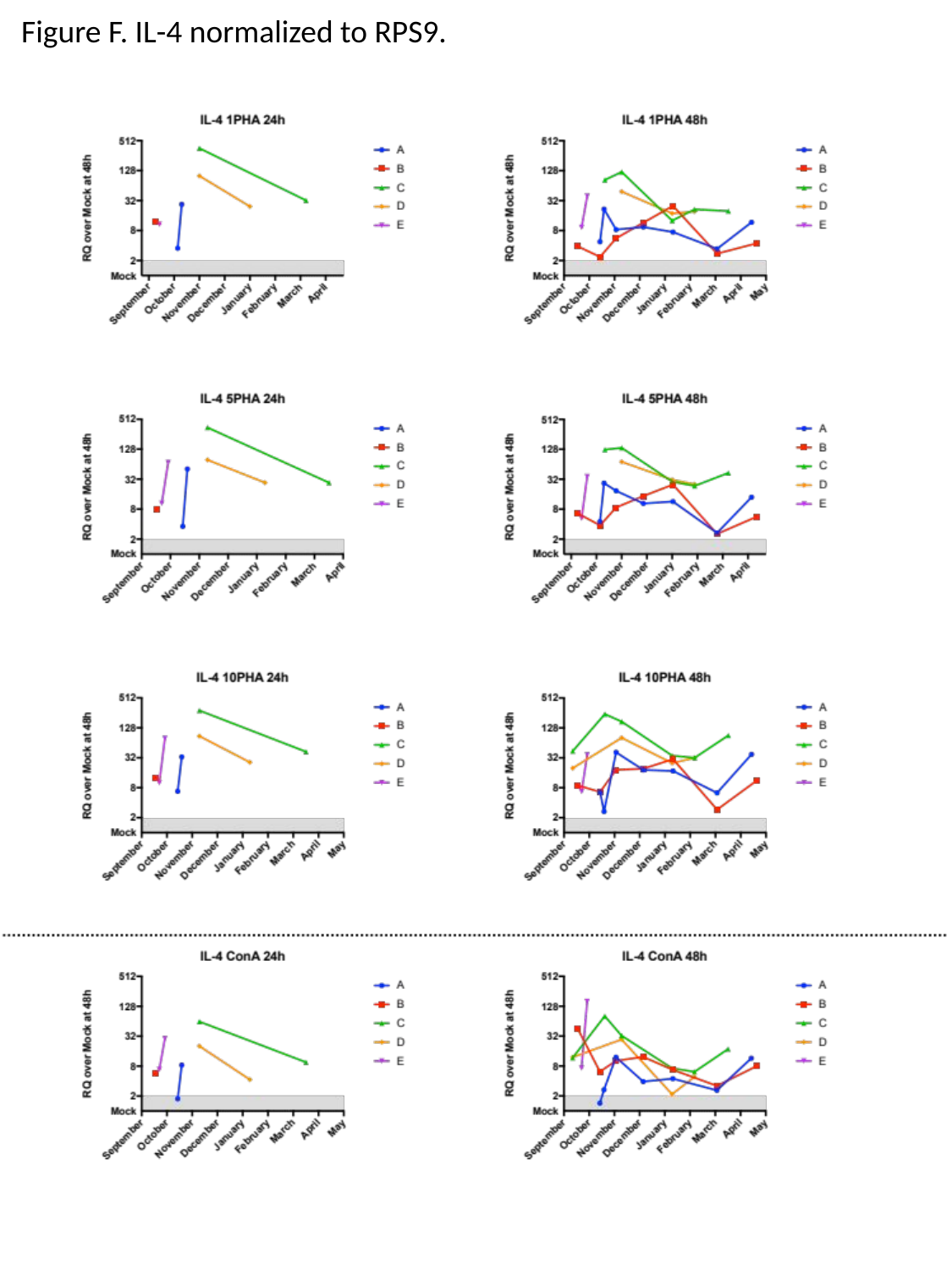

Figure F. IL-4 normalized to RPS9.

## Slide 7
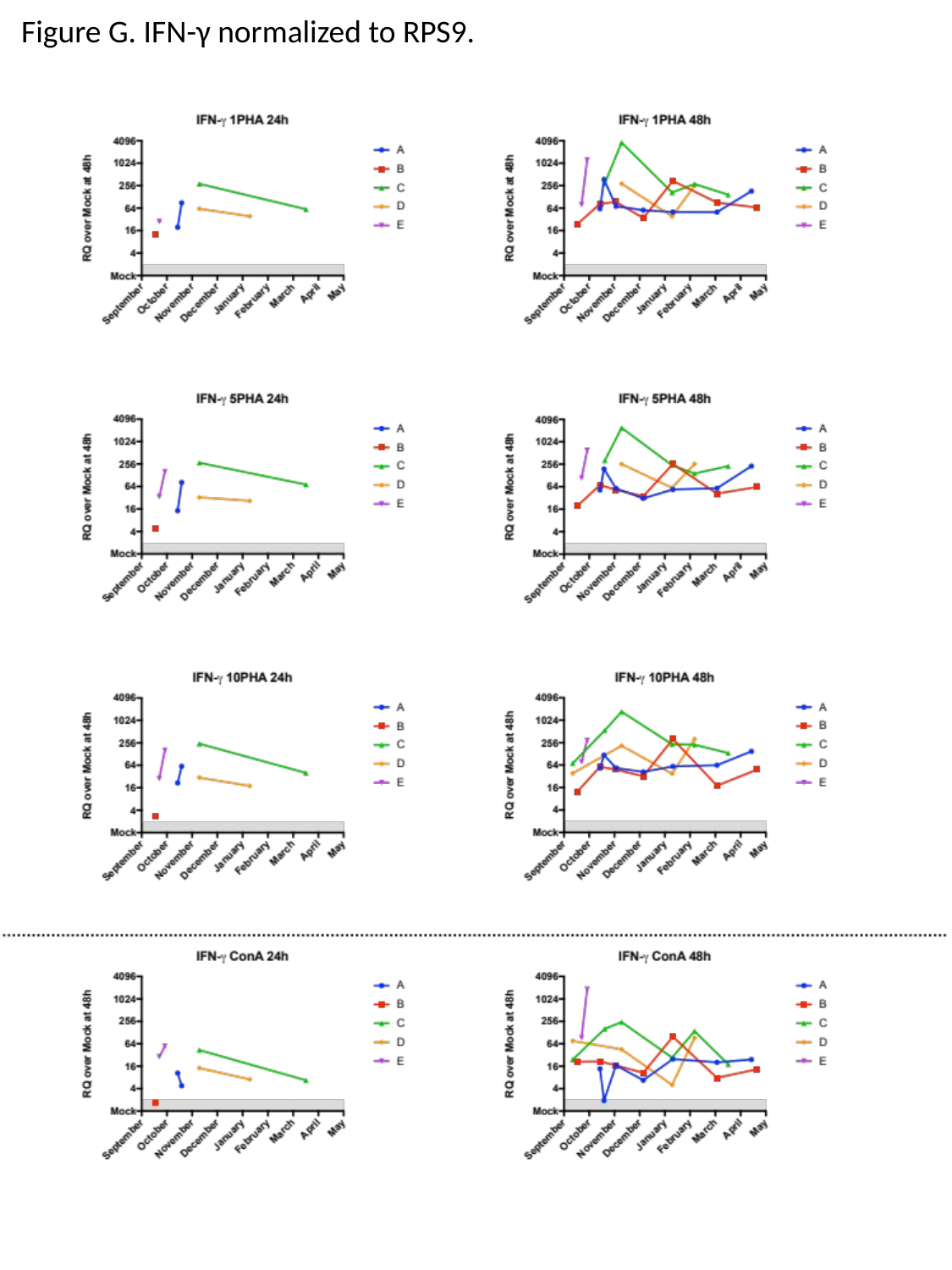

Figure G. IFN-γ normalized to RPS9.

## Slide 8
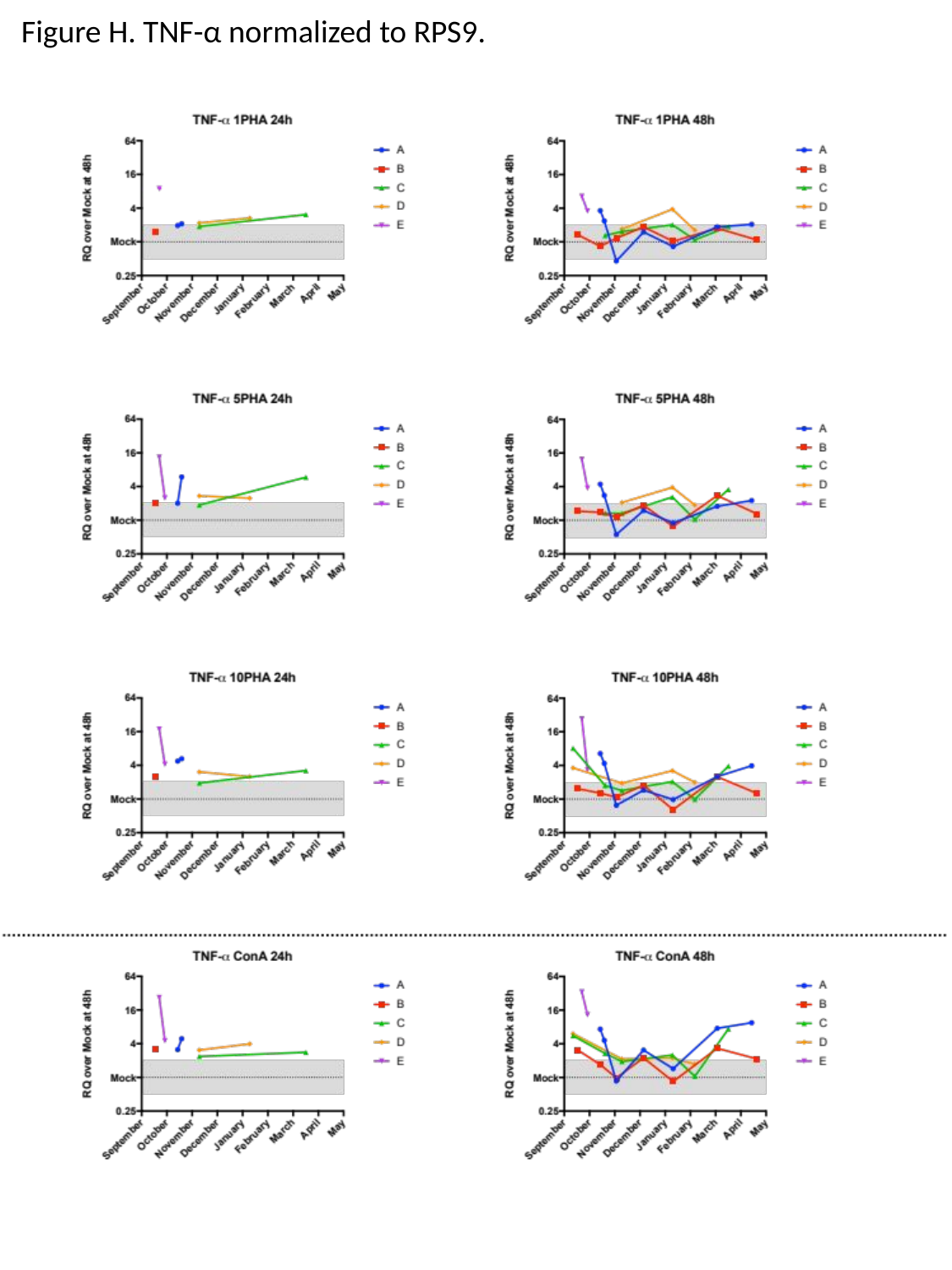

Figure H. TNF-α normalized to RPS9.

## Slide 9
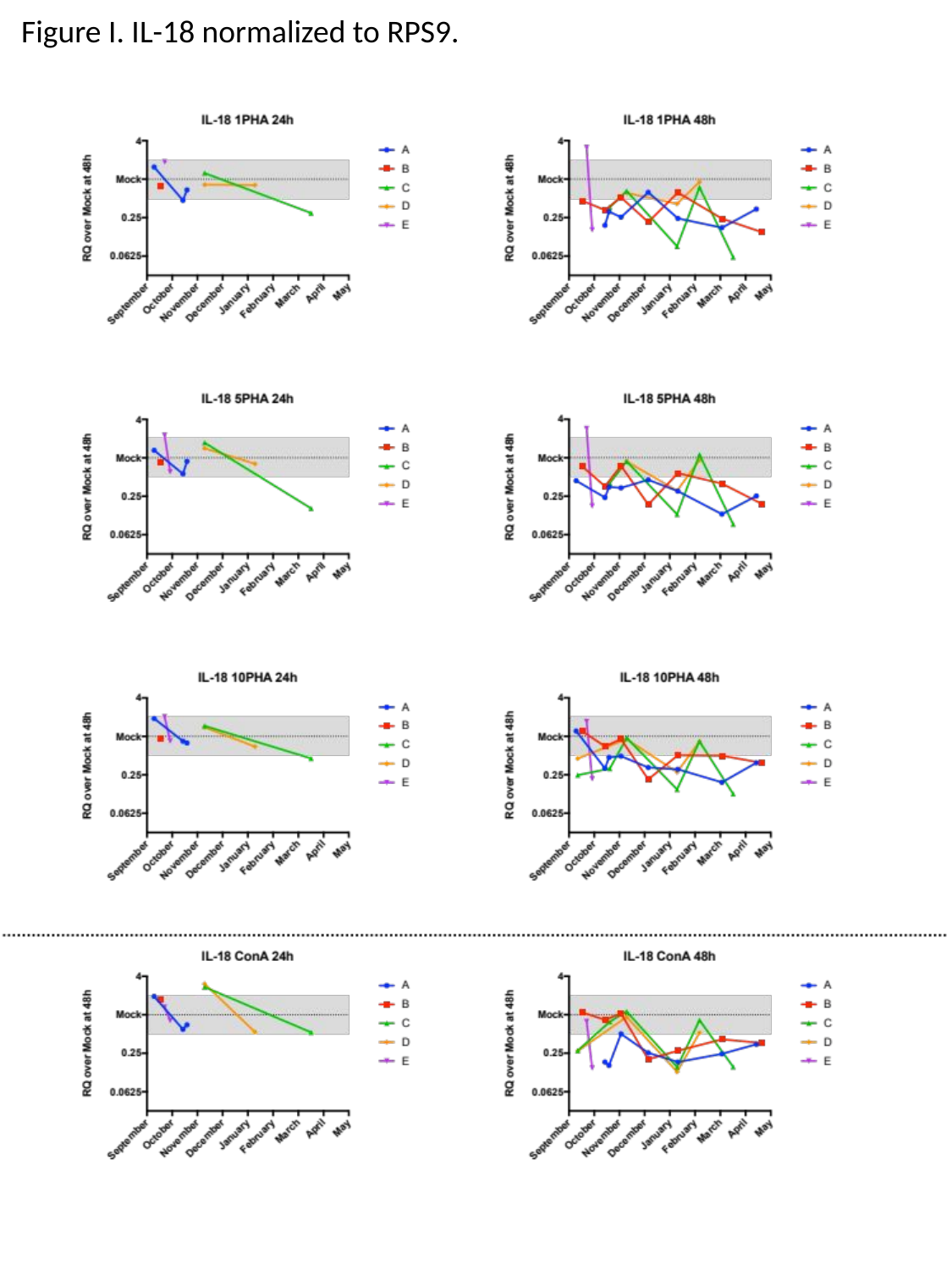

Figure I. IL-18 normalized to RPS9.

## Slide 10
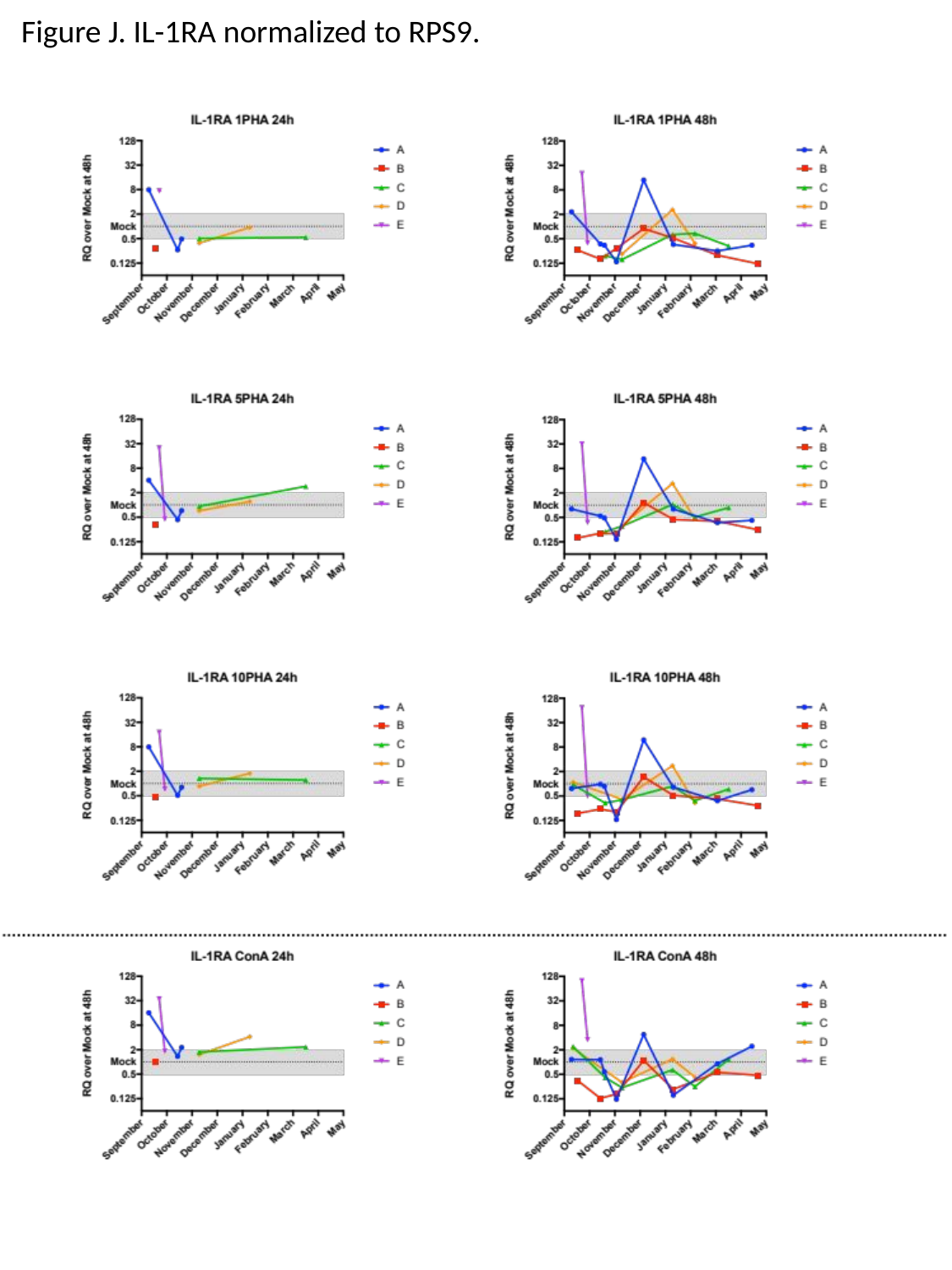

Figure J. IL-1RA normalized to RPS9.

## Slide 11
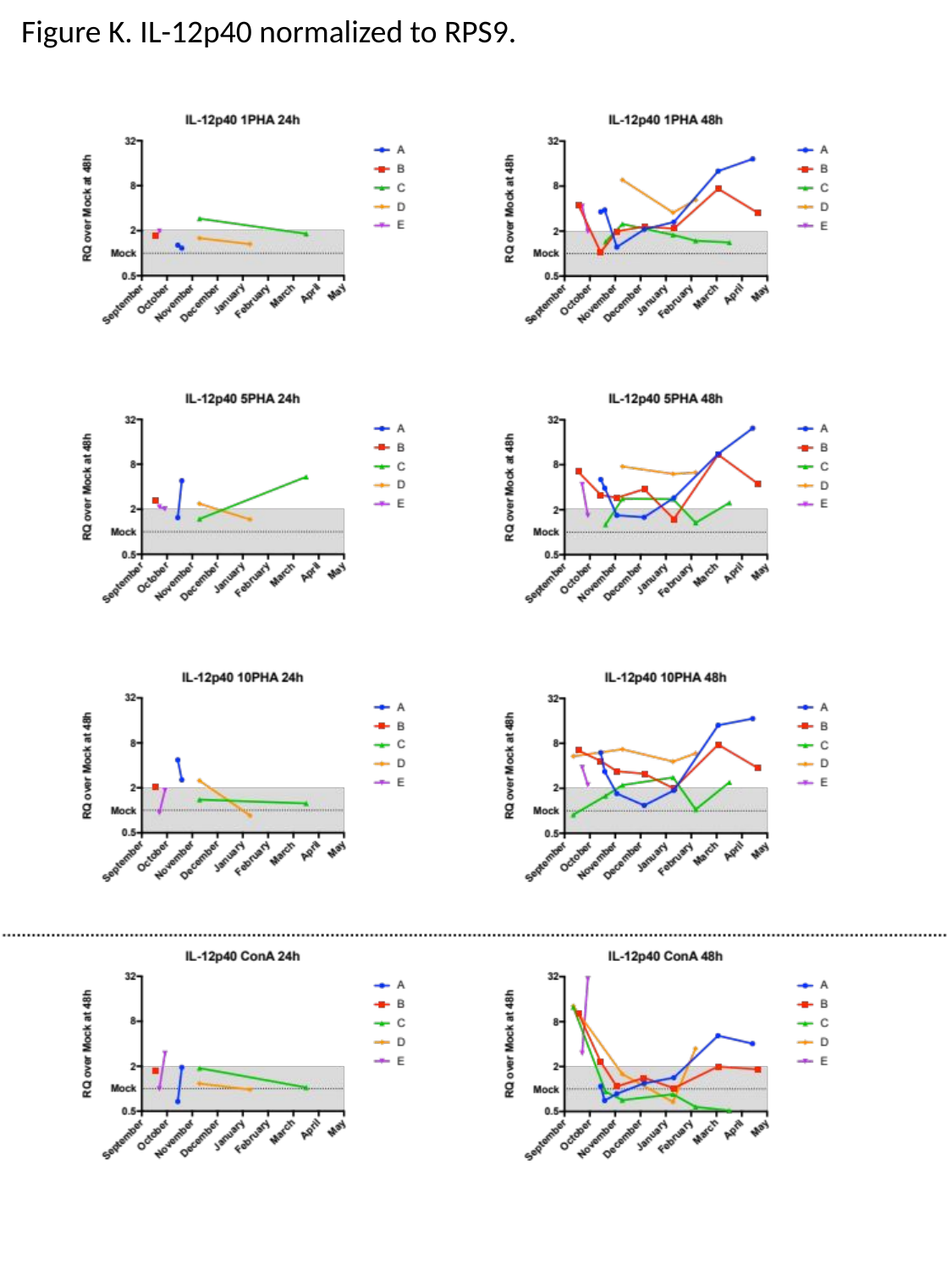

Figure K. IL-12p40 normalized to RPS9.

## Slide 12
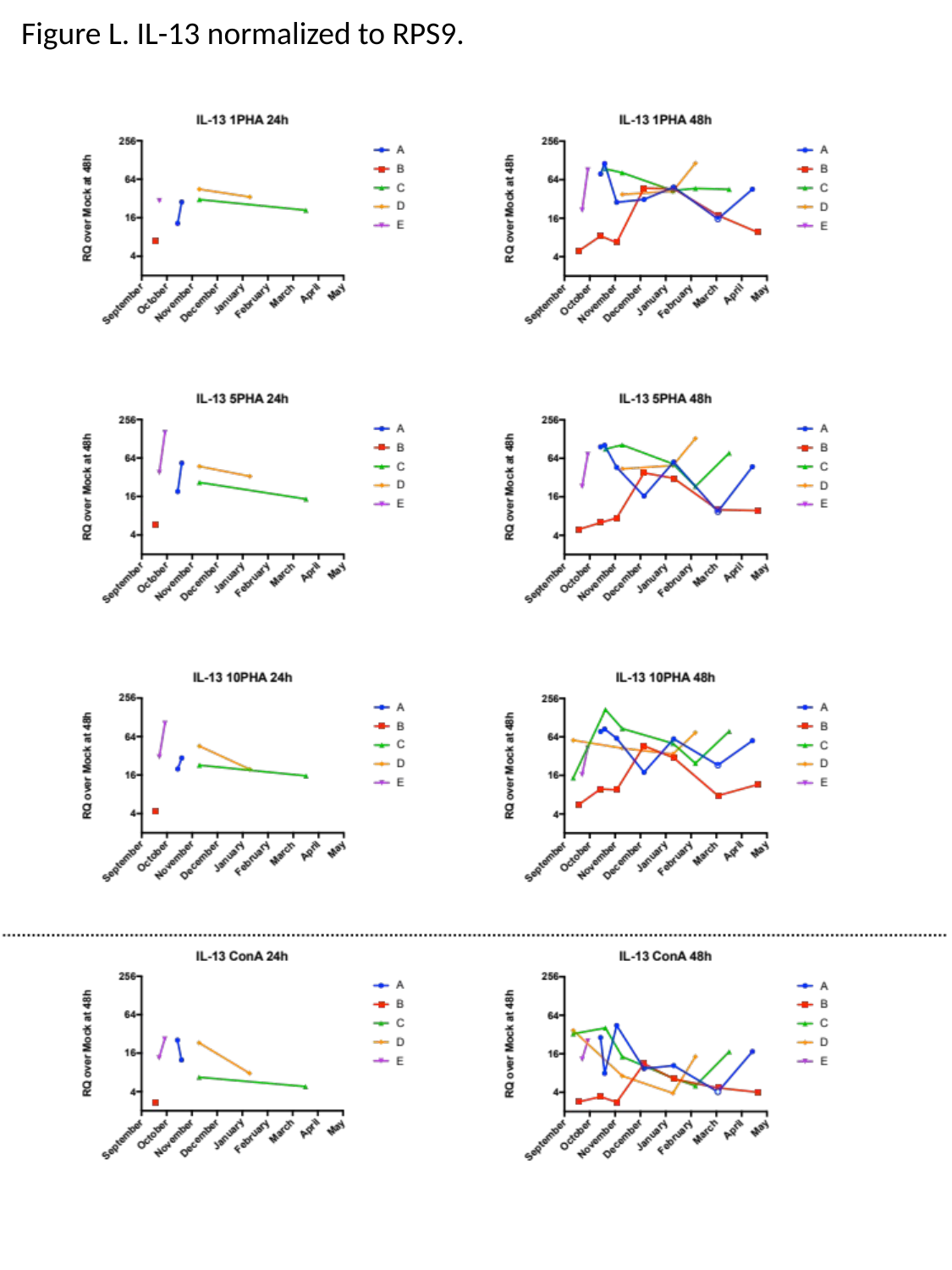

Figure L. IL-13 normalized to RPS9.

## Slide 13
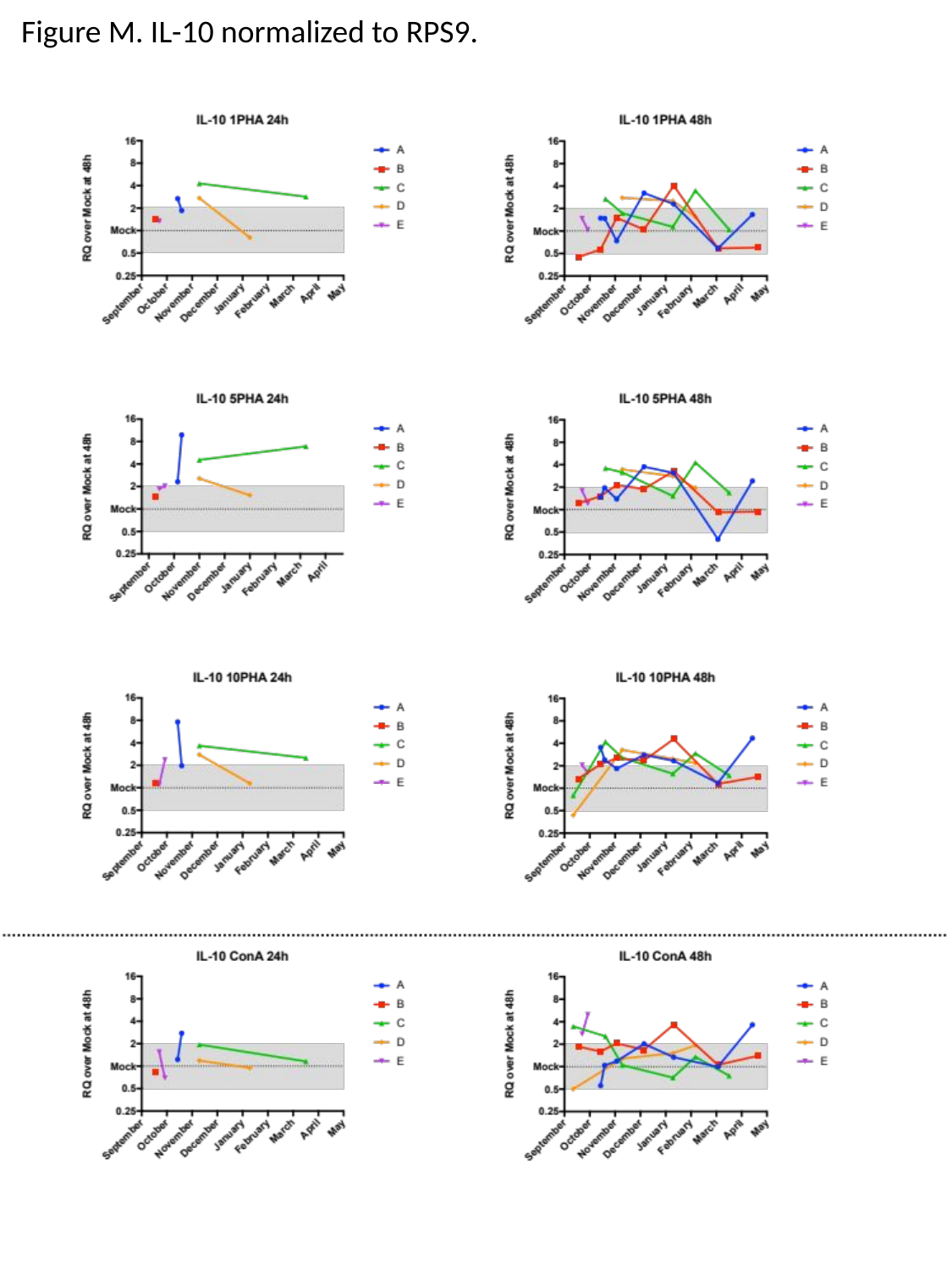

Figure M. IL-10 normalized to RPS9.

## Slide 14
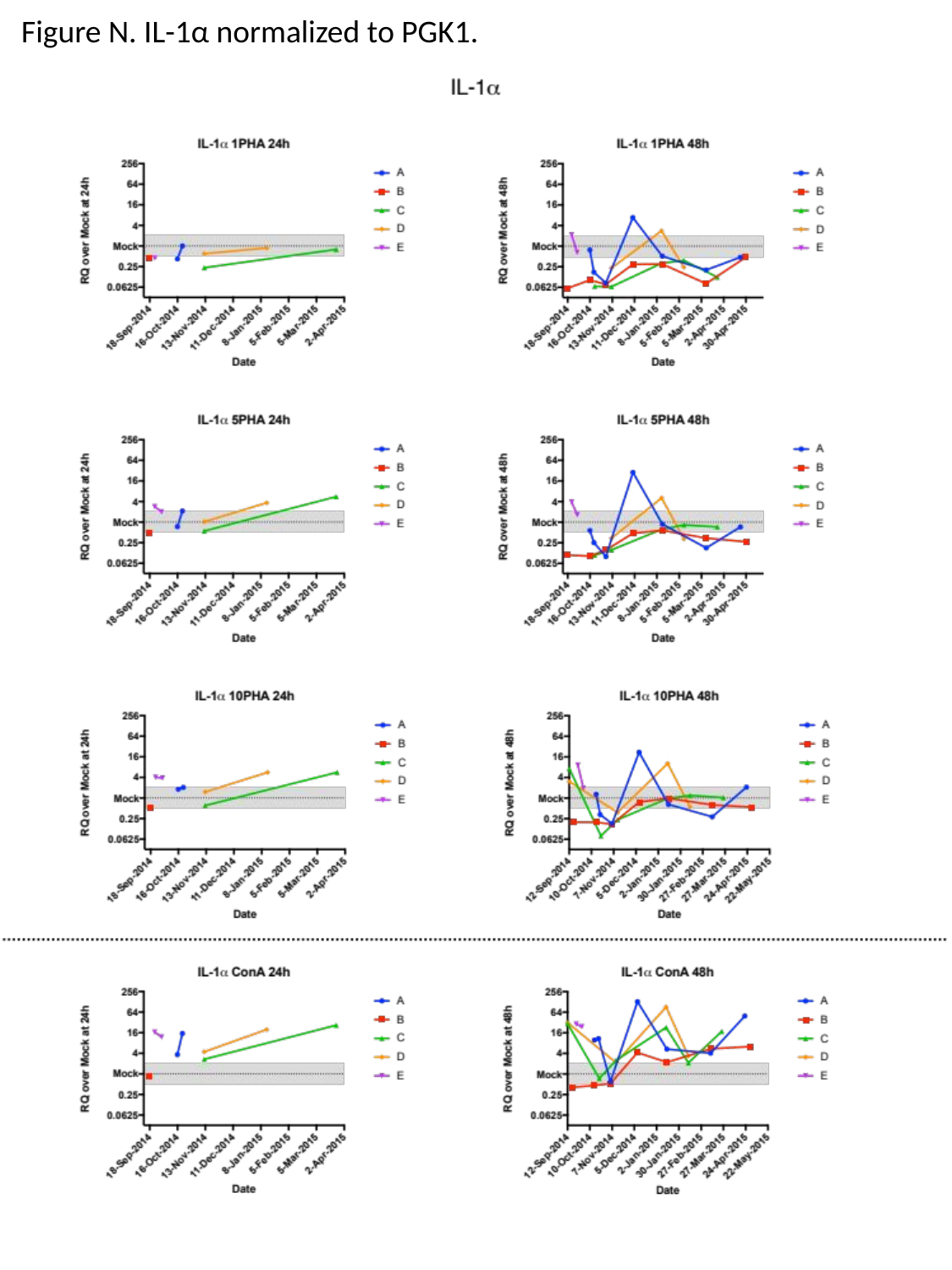

Figure N. IL-1α normalized to PGK1.

## Slide 15
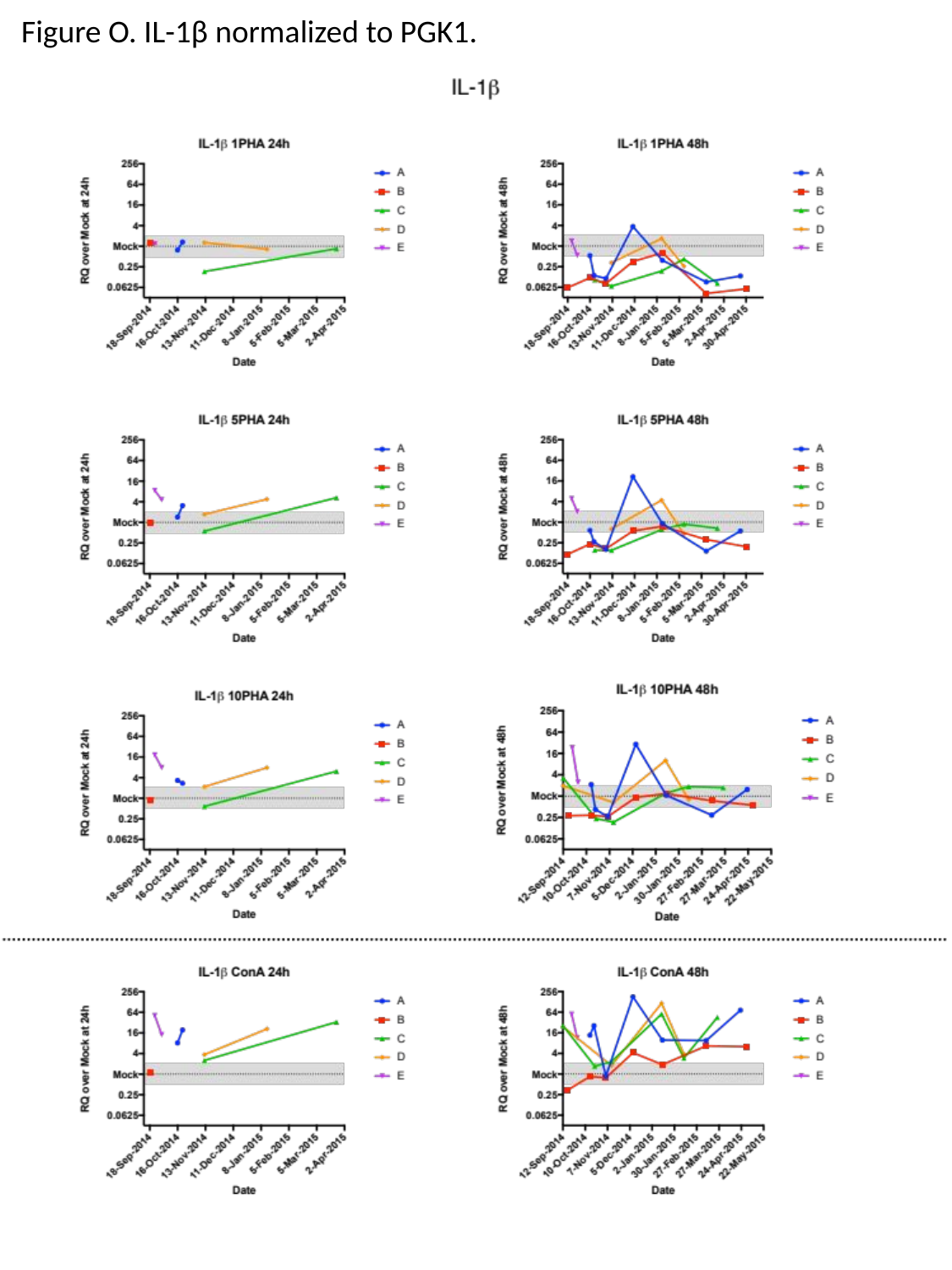

Figure O. IL-1β normalized to PGK1.

## Slide 16
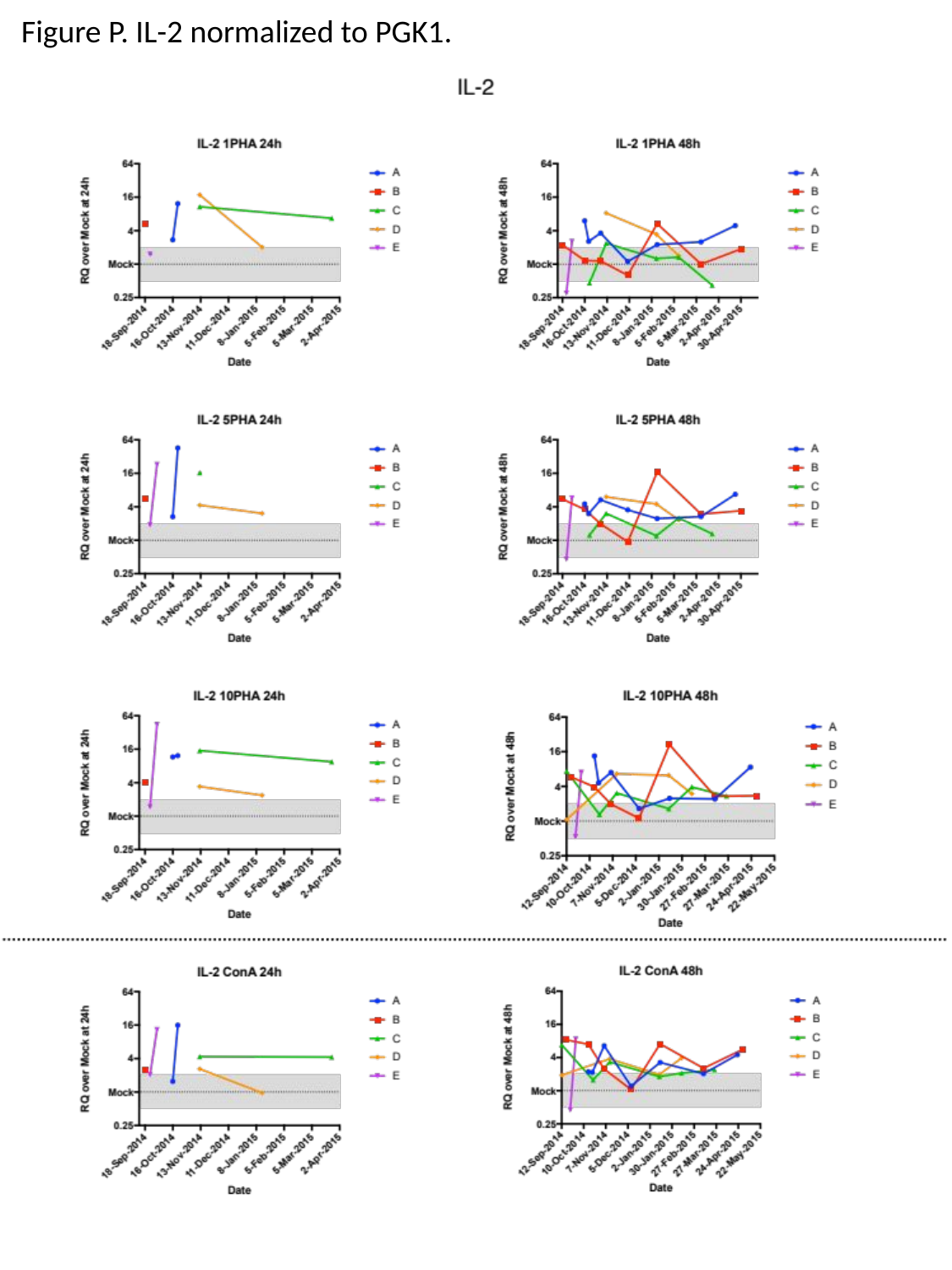

Figure P. IL-2 normalized to PGK1.

## Slide 17
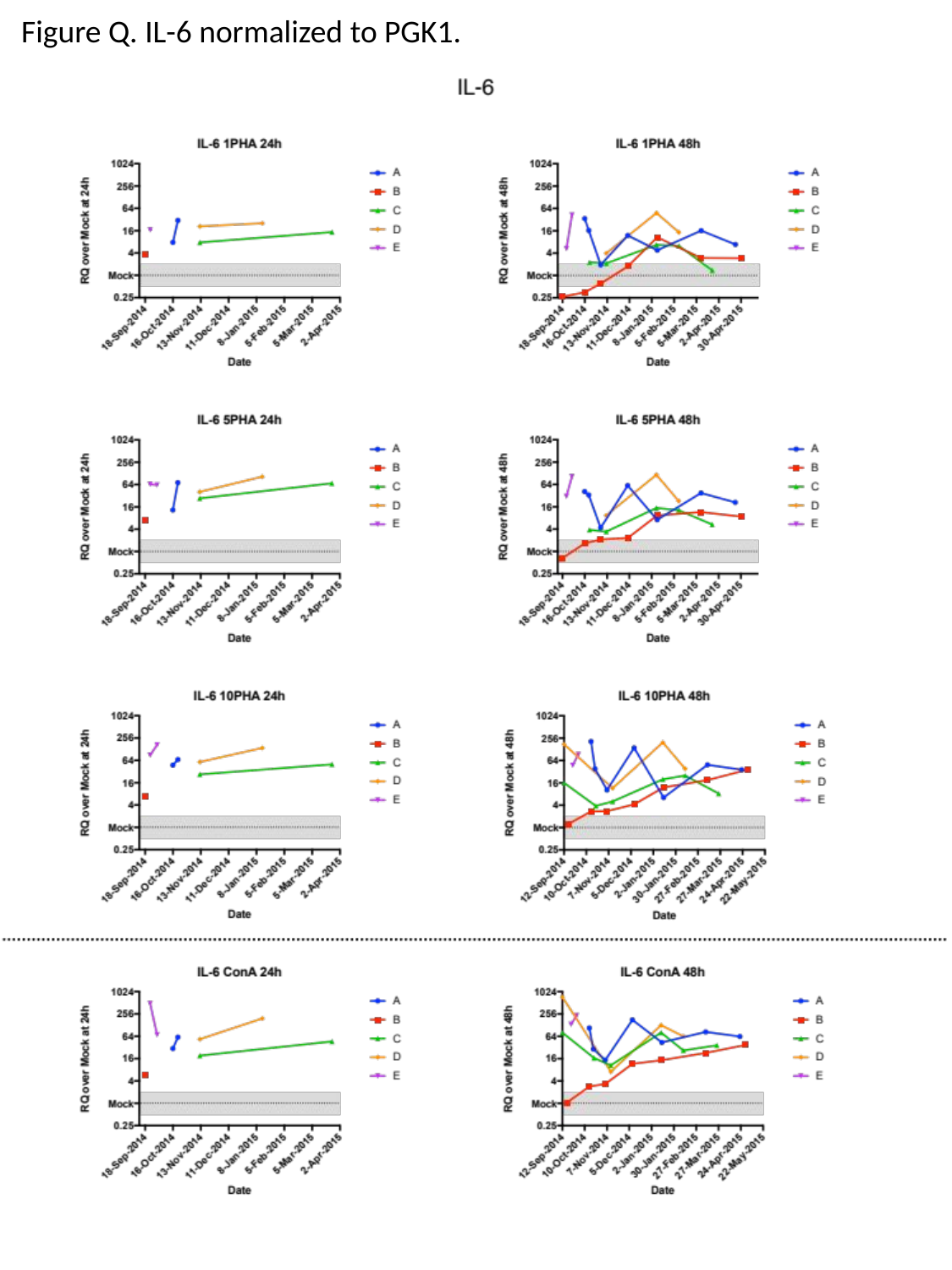

Figure Q. IL-6 normalized to PGK1.

## Slide 18
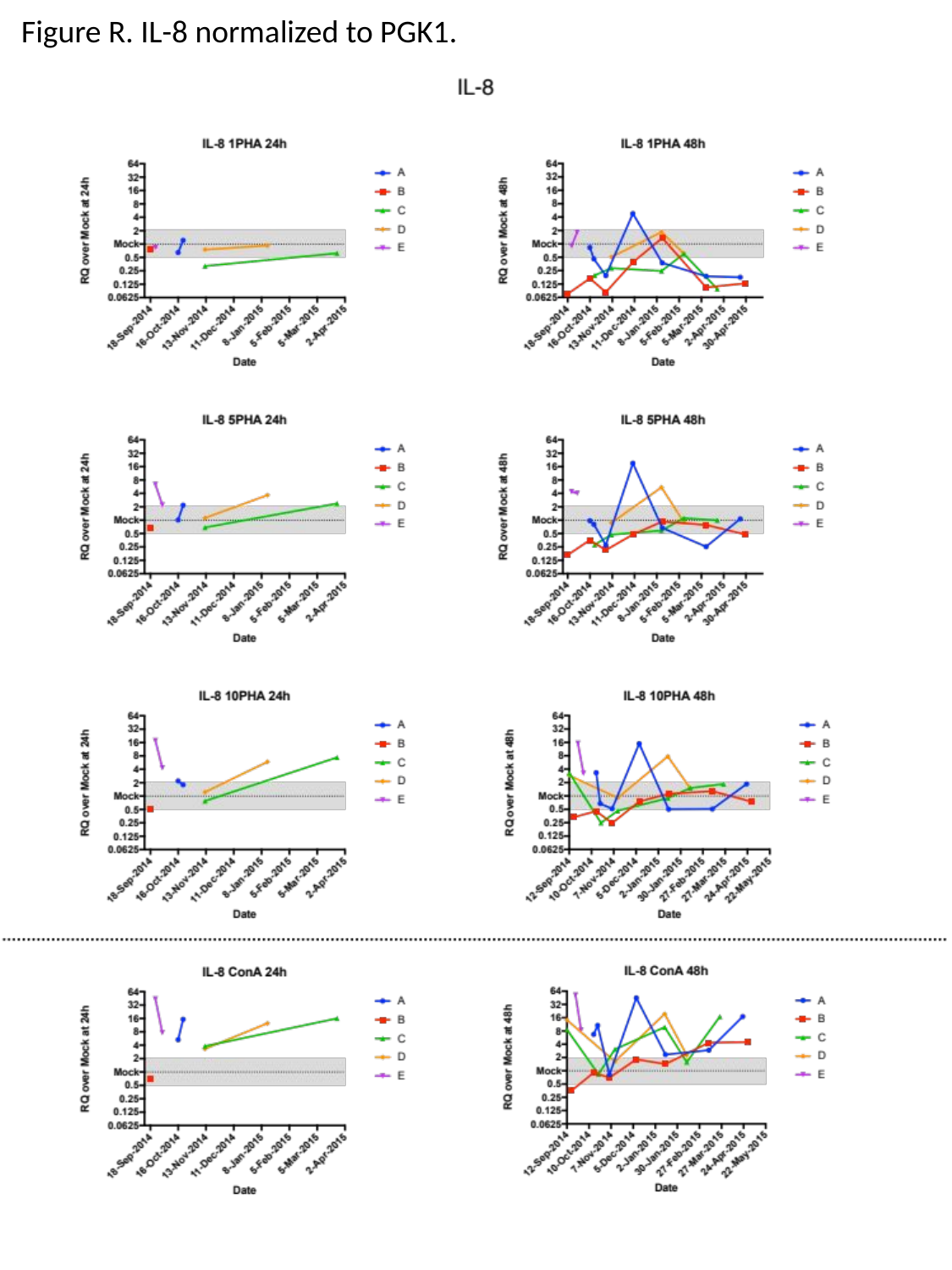

Figure R. IL-8 normalized to PGK1.

## Slide 19
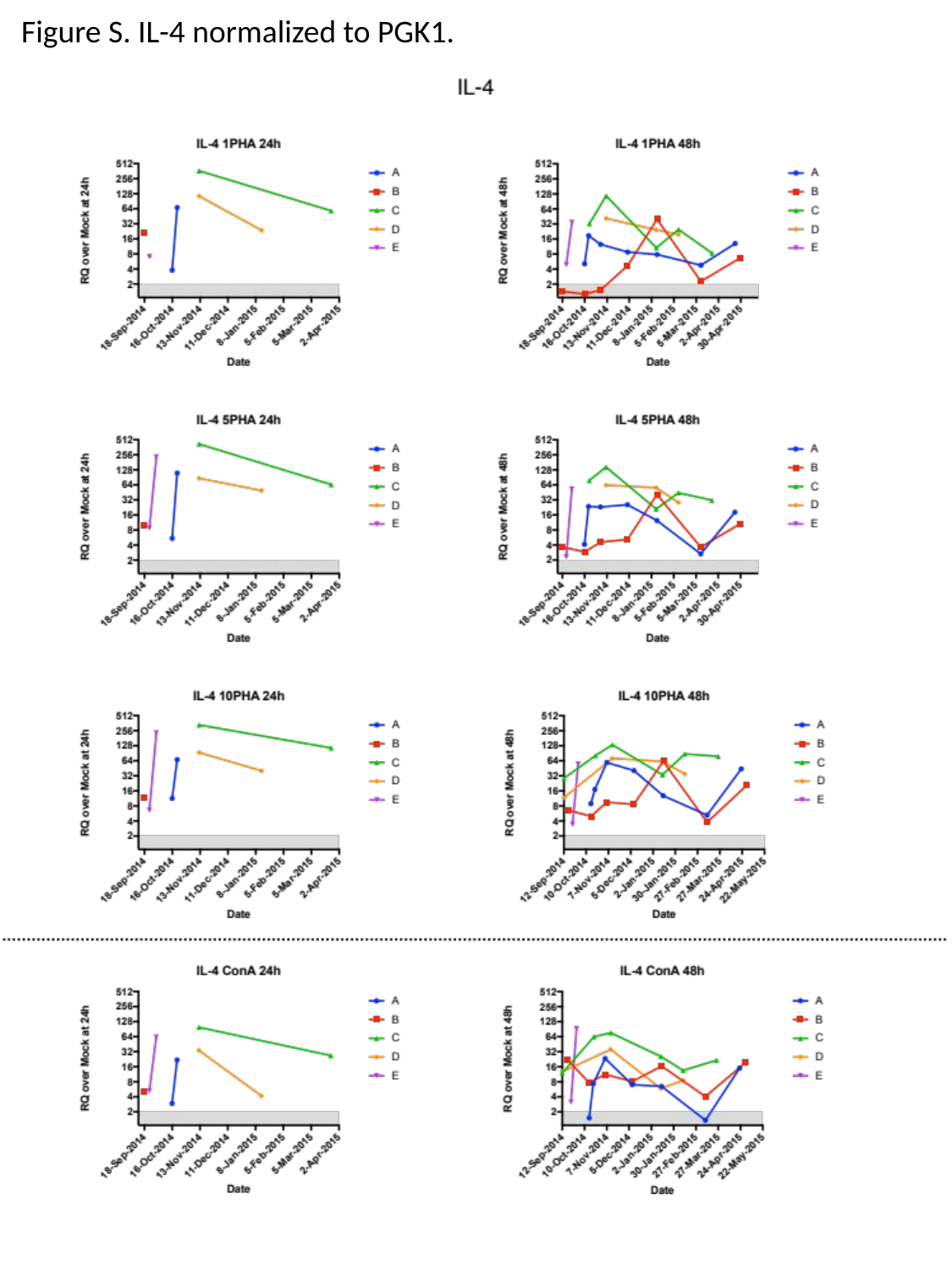

Figure S. IL-4 normalized to PGK1.

## Slide 20
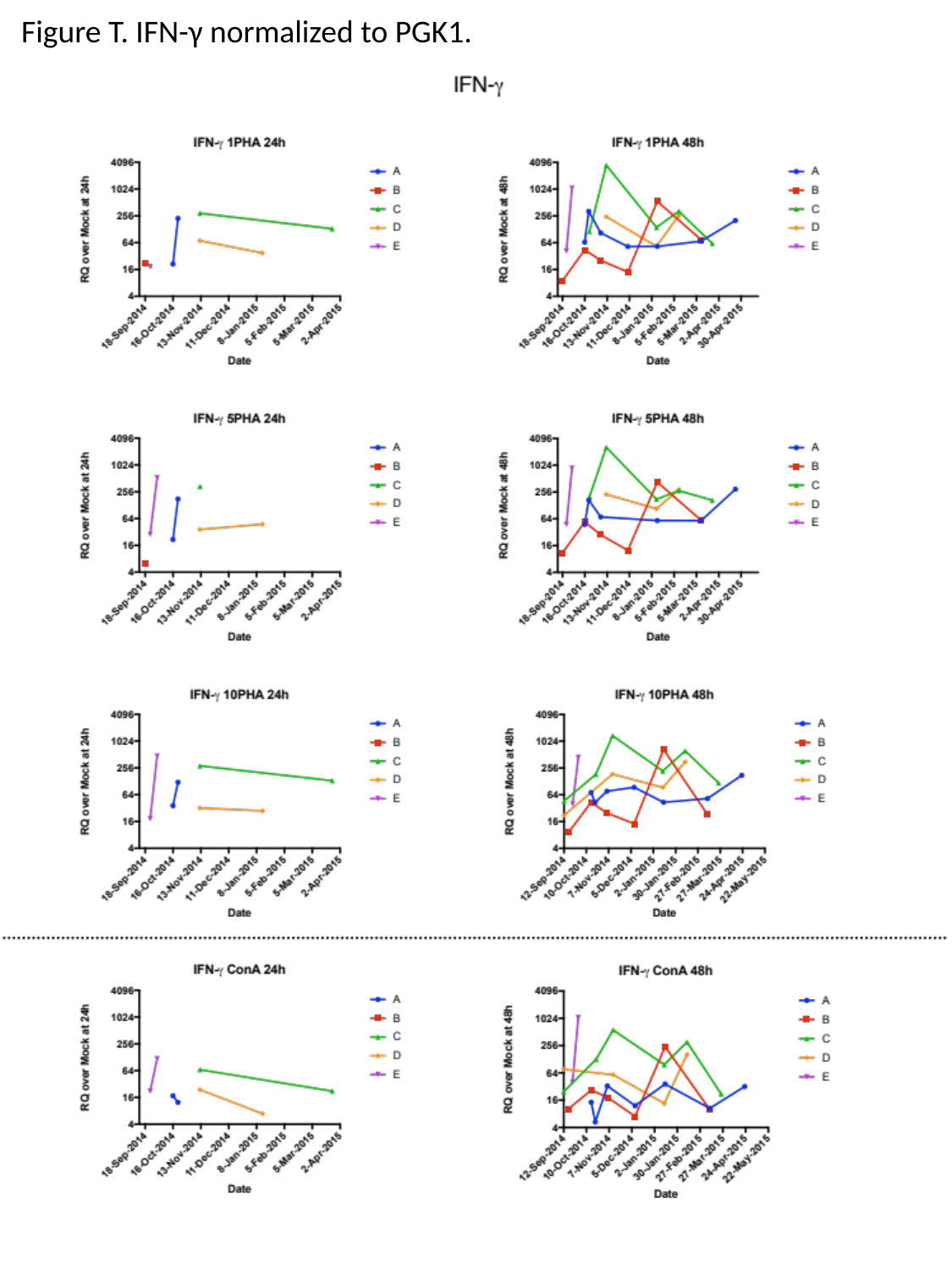

Figure T. IFN-γ normalized to PGK1.

## Slide 21
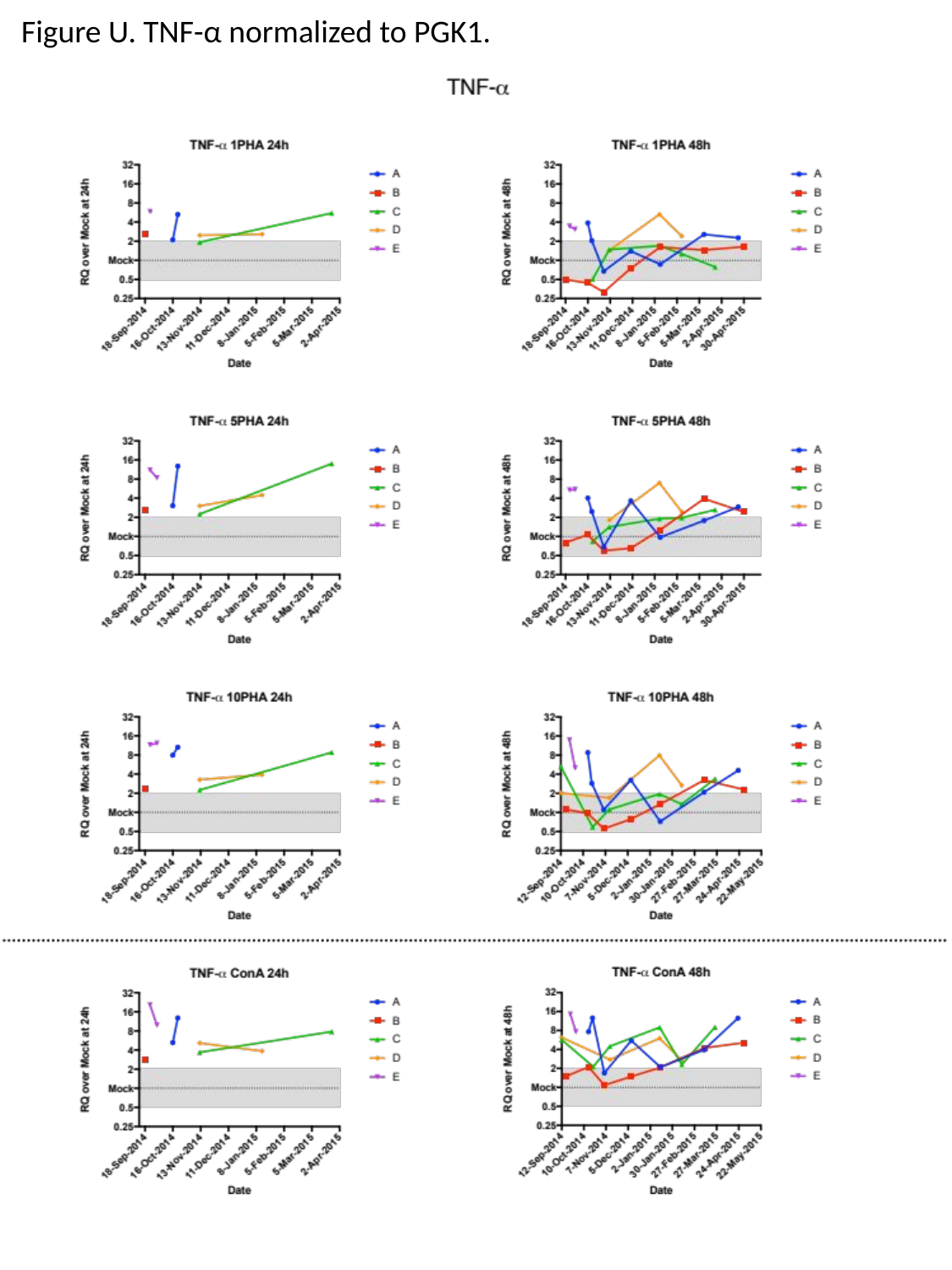

Figure U. TNF-α normalized to PGK1.

## Slide 22
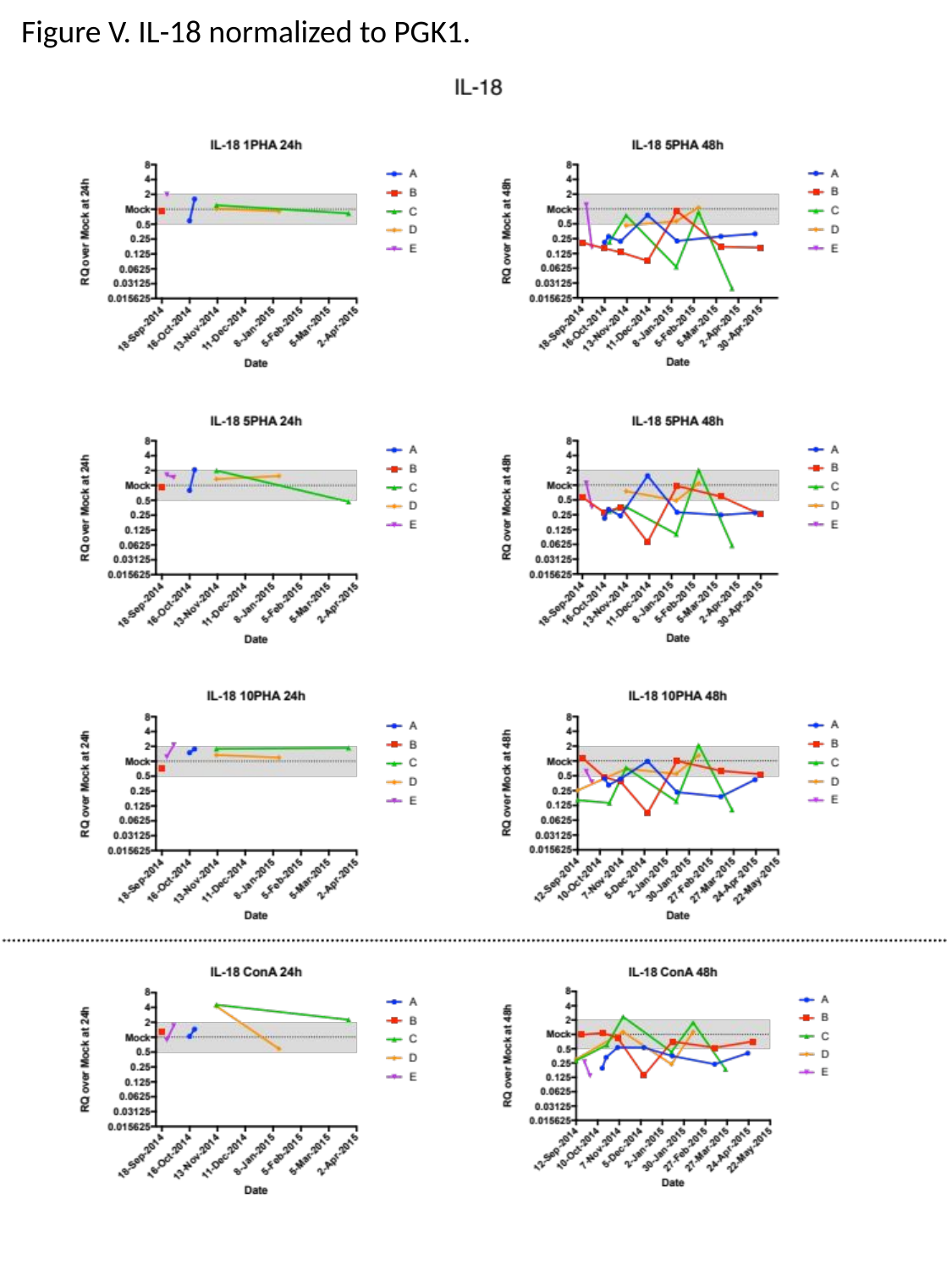

Figure V. IL-18 normalized to PGK1.

## Slide 23
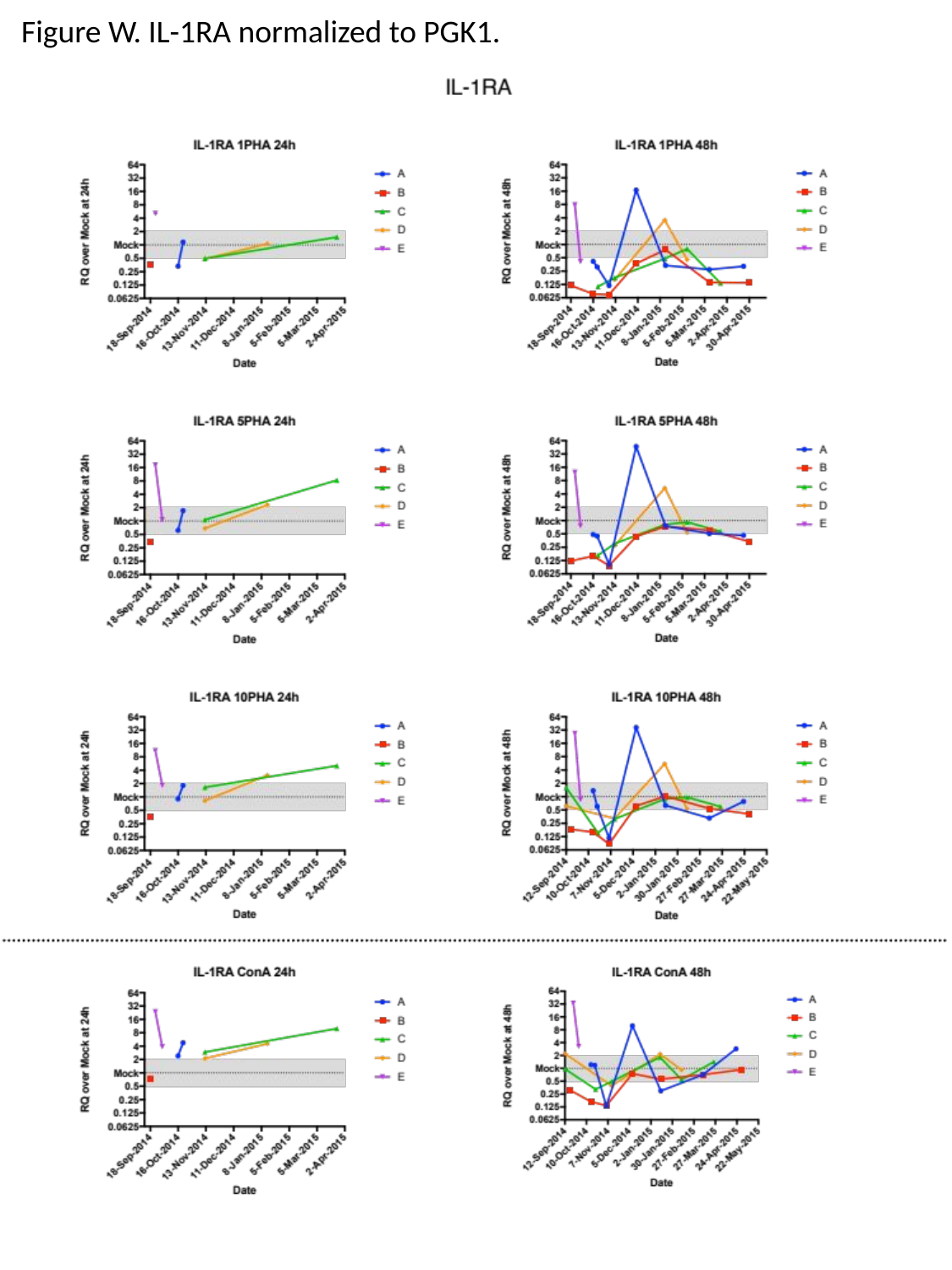

Figure W. IL-1RA normalized to PGK1.

## Slide 24
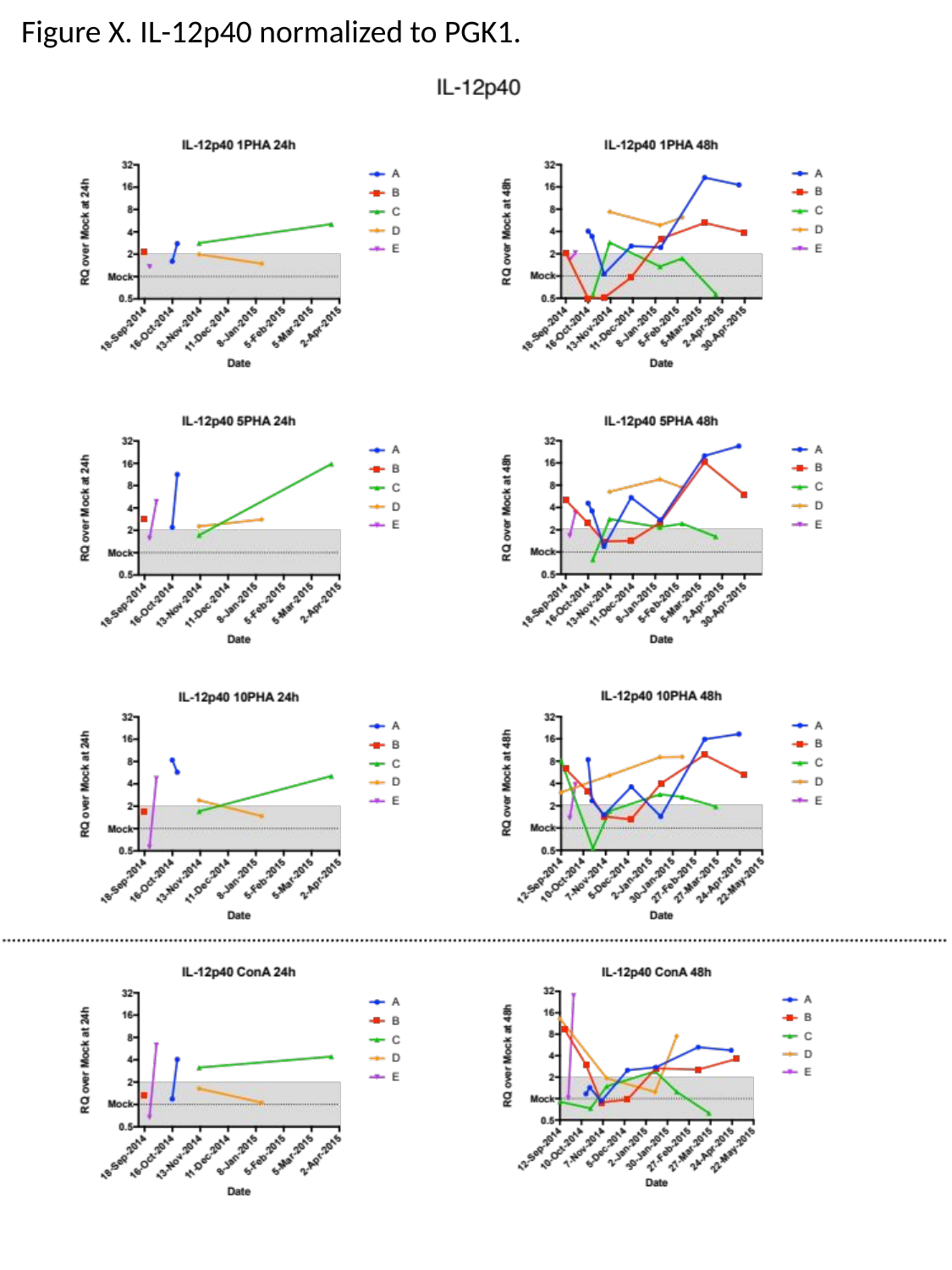

Figure X. IL-12p40 normalized to PGK1.

## Slide 25
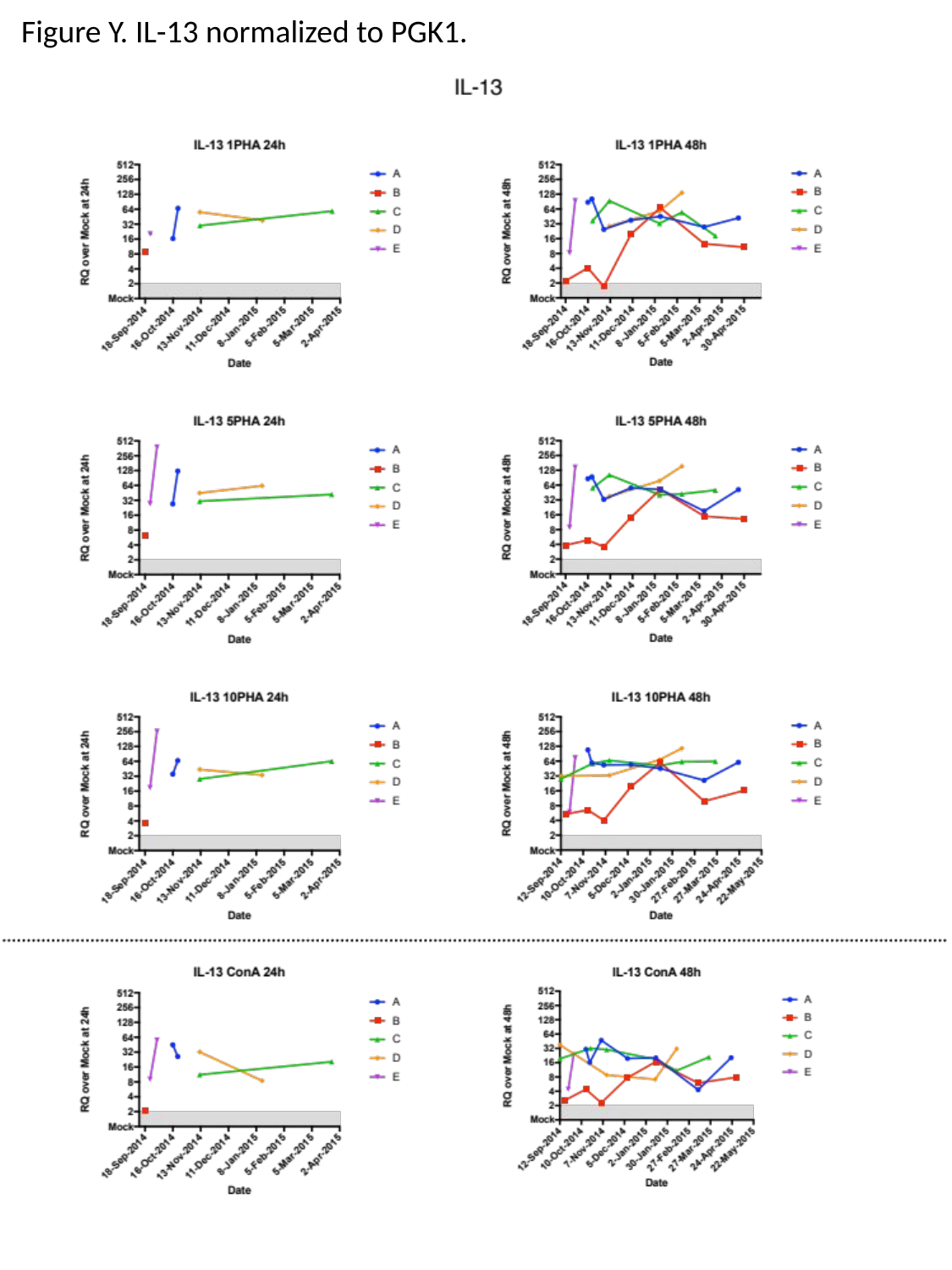

Figure Y. IL-13 normalized to PGK1.

## Slide 26
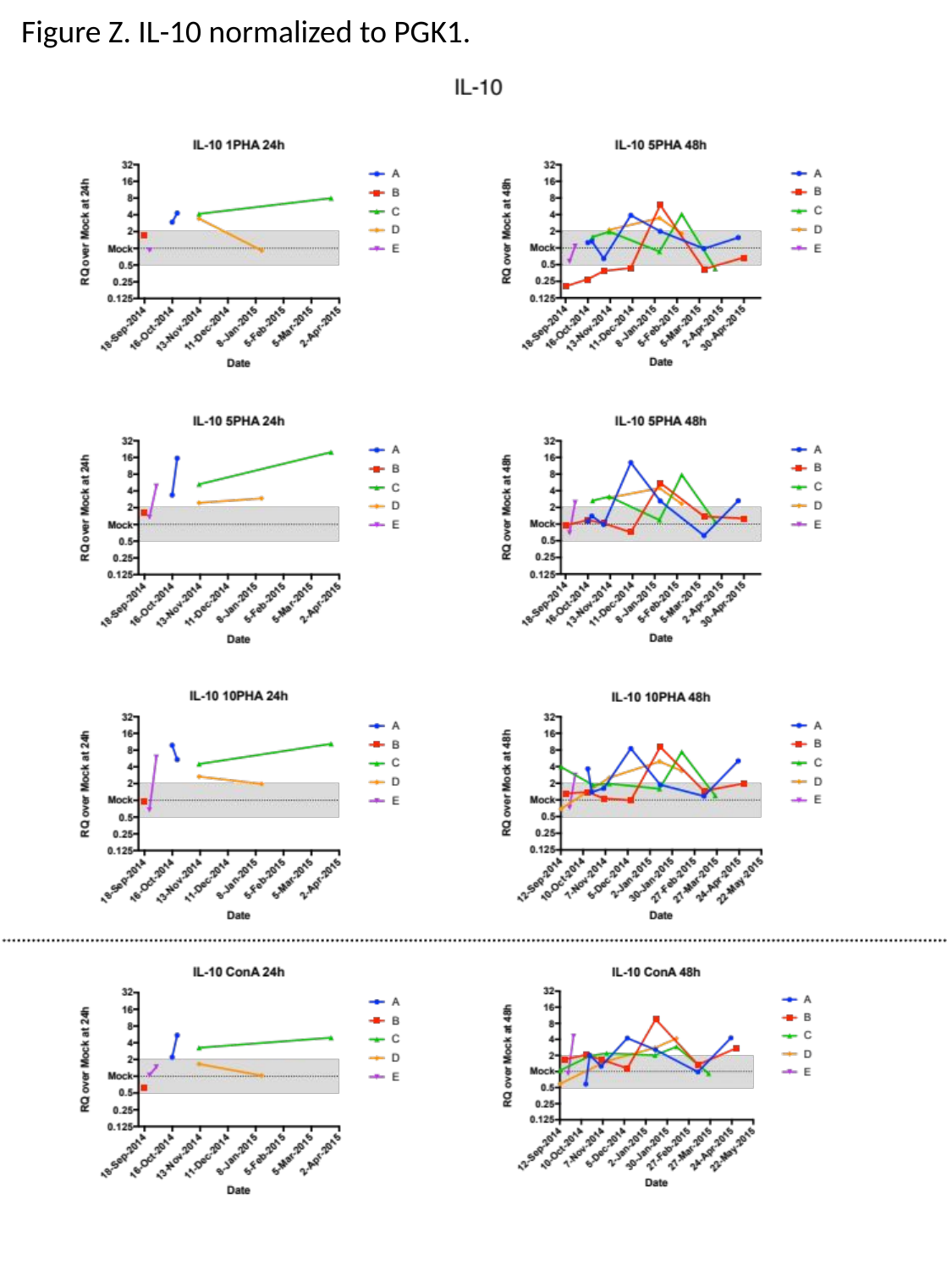

Figure Z. IL-10 normalized to PGK1.
