# Supplementary material for: Monitoring bottlenose dolphin leukocyte cytokine mRNA responsiveness by qPCR
Source: PLoS One. 2017 Dec 22;12(12):e0189437. doi: 10.1371/journal.pone.0189437 (PMC5741220; doi:10.1371/journal.pone.0189437)
Supplement: S2 Appendix — Normal RQ values for each cytokine and stimulus were pooled into two groups. Three to four samples from dolphin A were pooled with 4 samples from dolphin D. Similarly, 5 samples from dolphin B were pooled with one to two samples from dolphin C. RQ values normalized to RPS9 are plotted against sampling date. Shaded area indicates range from 0.5–2 RQ considered no different from mock. (PPTX) [file pone.0189437.s002.pptx]

## Slide 1
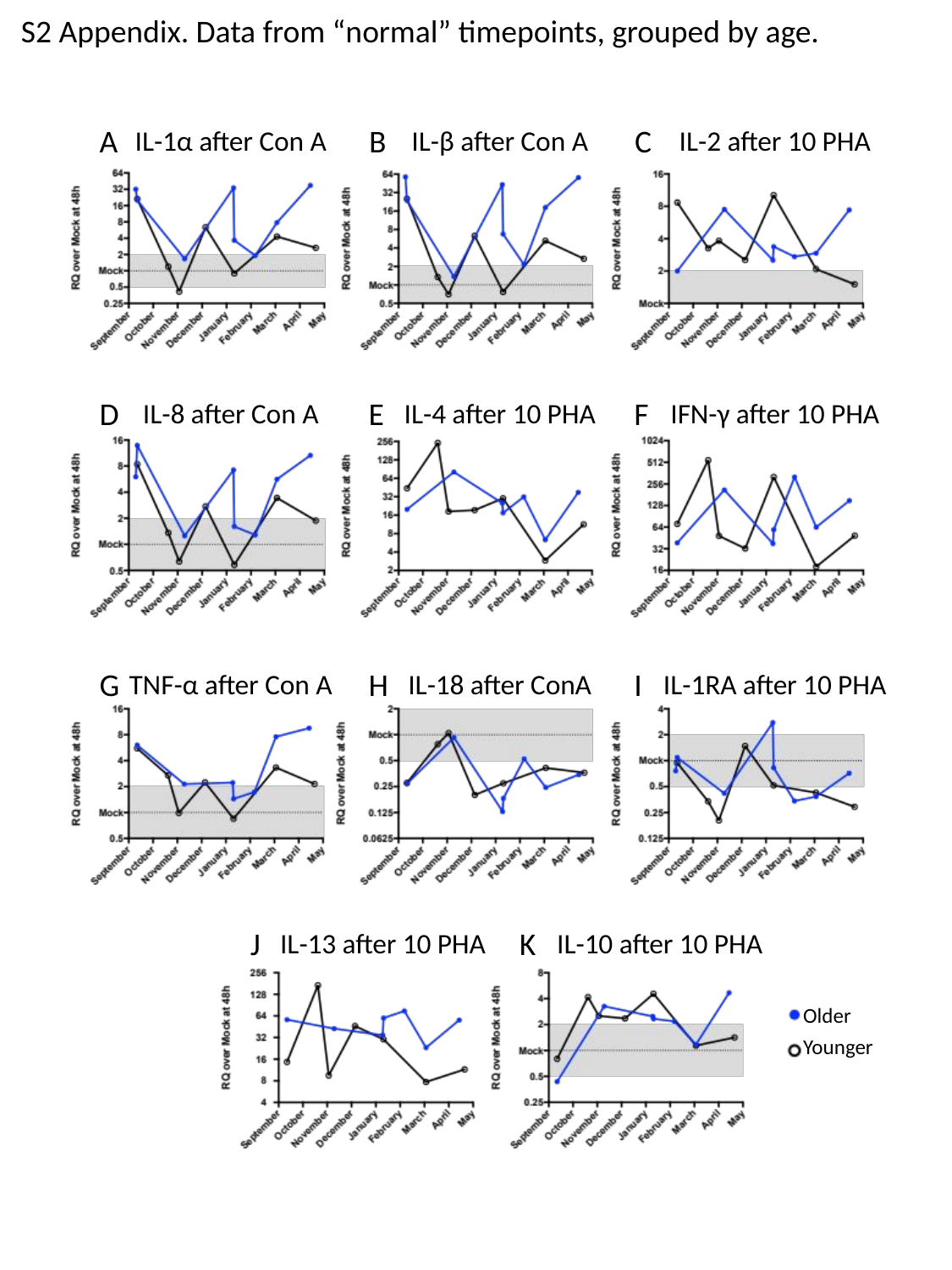

S2 Appendix. Data from “normal” timepoints, grouped by age.
A
B
C
IL-1α after Con A
IL-β after Con A
IL-2 after 10 PHA
D
E
F
IL-8 after Con A
IL-4 after 10 PHA
IFN-γ after 10 PHA
G
H
I
TNF-α after Con A
IL-18 after ConA
IL-1RA after 10 PHA
J
K
IL-13 after 10 PHA
IL-10 after 10 PHA
Older
Younger
