# Supplementary figures and images for: Monitoring bottlenose dolphin leukocyte cytokine mRNA responsiveness by qPCR
Source: PLoS One. 2017 Dec 22;12(12):e0189437. doi: 10.1371/journal.pone.0189437 (PMC5741220; doi:10.1371/journal.pone.0189437)

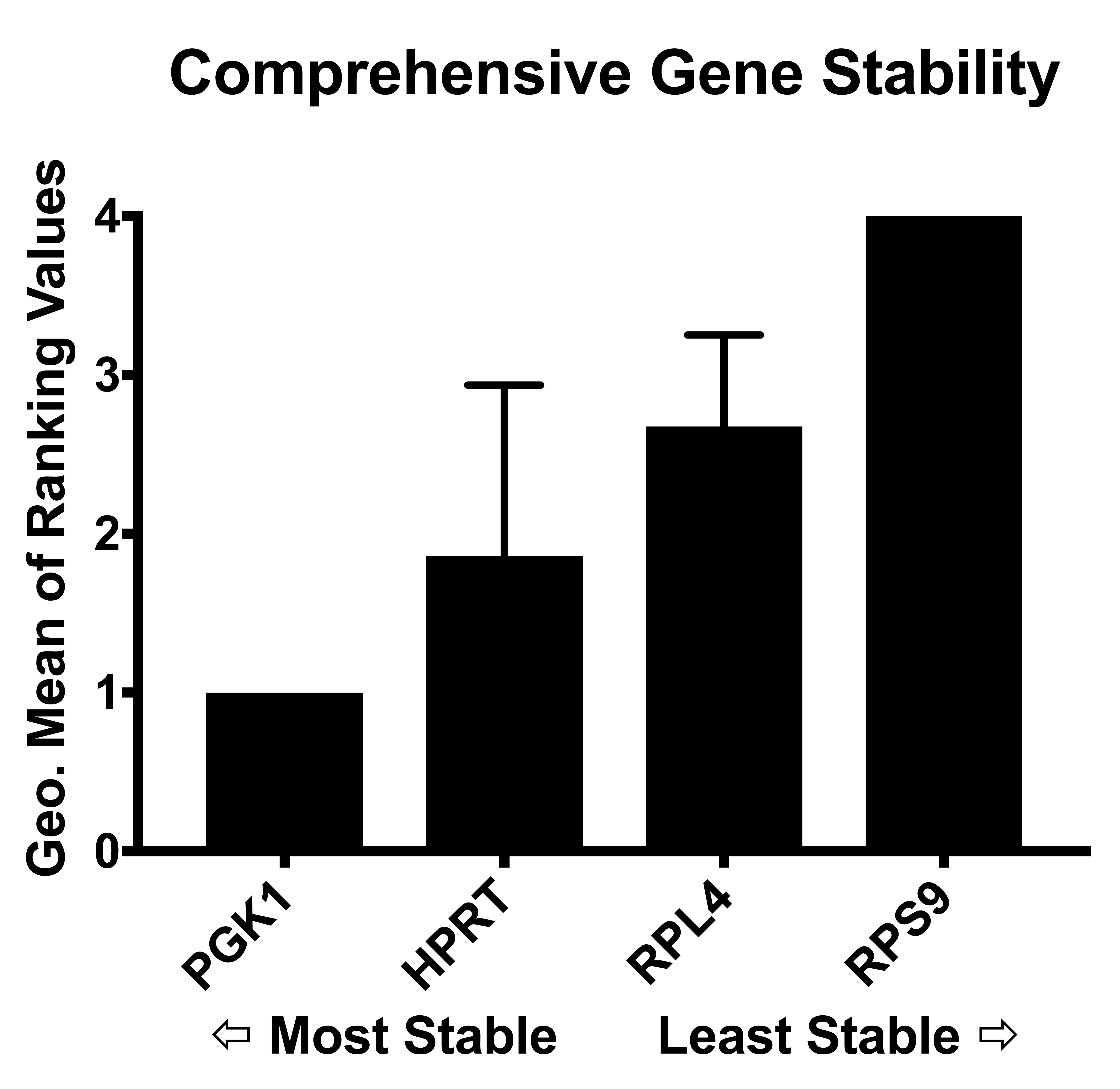

Supplement: S1 Fig — The three housekeeping genes determined to be most stable by Chen, et al. [20] (PGK1, RPL4 and HPRT) were compared with RPS9. The primers described by Chen, et al. [20] were used for PGK1, RPL4 and HPRT. Our own RPS9 primers were used for RPS9 (Table 2). The CtCorr (described in [20]) for these genes were determined for 24–37 randomly selected cDNA samples. The CtCorr values were compared using the free online program RefFinder (http://leonxie.esy.es/RefFinder/?type=reference#). This program uses four different computational programs to rank and weight the stability of housekeeping genes. RefFinder then calculates the geometric mean of the weights given by the four programs. The resulting geometric means and geometric standard deviations of the ranking values for each housekeeping gene are shown. (TIF) [file pone.0189437.s003.tif]
